# Burden of Acute-Care Hospitalization for Community-Acquired Pneumonia in Canadian Adults Aged 50 Years or Older: Focusing on Most Responsible Diagnosis Tells Only Part of the Story

**DOI:** 10.3390/vaccines11040748

**Published:** 2023-03-28

**Authors:** Ana Gabriela Grajales Beltrán, Derek Lytle, Jelena Vojicic, Prerna Grover, Lidija Latifovic, Shane Golden, Juejing Ling, Brad Millson, Alejandro Cane

**Affiliations:** 1 Vaccines Medical Affairs, Pfizer Canada, Kirkland, QC H9J 2M5, Canada; 2 Market Access, Pfizer Canada, Kirkland, QC H9J 2M5, Canada; 3 Real-World Solutions, IQVIA Canada, Mississauga, ON L5N 6A4, Canada; 4 Vaccines Medical and Scientific Affairs, North America, Pfizer Inc., Collegeville, PA 19426, USA

**Keywords:** community-acquired pneumonia, Canada, real world, most responsible diagnosis (MRDx), other than most responsible diagnosis (ODx), hospitalization, costs

## Abstract

The burden of all-cause community-acquired pneumonia (CAP), including pneumococcal pneumonia, is typically estimated using ICD codes where pneumonia is coded as the most responsible diagnosis (MRDx). Pneumonia may also be coded as other than most responsible diagnosis (ODx) based on administrative and reimbursement criteria. Analyses including pneumonia as MRDx only likely underestimate hospitalized CAP incidence. The aim of this study was to estimate the burden of hospitalized all-cause CAP in Canada and to assess the contribution of ODx-coded cases to the overall disease burden. This longitudinal retrospective study obtained data from the Canadian Institutes of Health Information (CIHI) for adults 50+ years hospitalized for CAP between 1 April 2009 and 31 March 2019. Cases were identified as those where pneumonia was either diagnosis code type M (MRDx) or pre-admit comorbidity type 1 (ODx). Reported outcomes include pneumonia incidence rate, in-hospital mortality, hospital length of stay, and cost. Outcomes were stratified by age group, case coding, and comorbidity. Between 2009–2010 and 2018–2019, CAP incidence increased from 805.66 to 896.94 per 100,000. During this time, 55–58% of cases had pneumonia coded as ODx. Importantly, these cases had longer hospital stays, higher in-hospital mortality, and higher cost of hospitalization. The burden of CAP remains substantial and is significantly greater than that estimated by solely focusing on MRDx-coded cases. Our findings have implications for policy decision making related to current and future immunization programs.

## 1. Introduction

Community-acquired pneumonia (CAP) is a significant cause of clinical burden and mortality worldwide [1]. The risk of developing CAP increases with age and in adults with chronic underlying medical conditions [2]. CAP is associated with acute and long-term outcomes that have considerable implications for the healthcare system [3]. The burden of in-hospital treatment of CAP, including pneumococcal CAP (pCAP), is linked to high costs, in particular in cases associated with comorbidities [4]. The number of hospitalizations due to all-cause CAP in Canada during 2009–2010 was estimated to be 347 per 100,000 persons overall and 1537 per 100,000 in adults older than 65 years, with an in-hospital mortality rate of 11.6% and 16.7%, respectively [5]. Hospitalized all-cause CAP costs the healthcare system hundreds of millions (CAD) annually, and this burden is expected to increase in the following decades [6]. In the United States (USA), the economic burden of CAP was estimated to be USD 17 billion annually, and adults hospitalized with CAP during 2012–2016 spent an average of 5.7 days in the hospital, with an average total cost of hospitalization of USD 17,736 [7]. In Canada, the all-cause pneumonia cost was estimated to be CAD 216.2 million in 2010, and the average cost was forecasted to largely increase due to an aging population, increase in average cost per case, and population growth [6].

The adequate assessment of CAP incidence poses a challenge due to several factors including lack of standardized case definition, variability in diagnostic methods, and, unlike for reportable diseases (such as invasive pneumococcal disease, IPD), the fact that there is no routine surveillance for CAP in most countries, Canada included [8]. In addition, most patients with CAP are treated in an outpatient setting, yet most of the estimates of CAP incidence are based on hospitalized cases. Incidence assessments of hospitalized CAP, ideally with the determination of etiology and clinical outcomes, are needed to better determine prevention strategies and resource allocation [9].

Administrative data are commonly used to estimate the incidence of hospitalized CAP, using ICD codes where pneumonia is coded as the most responsible diagnosis (MRDx). Since Canadian coding standards require prioritization of certain comorbidities, pneumonia, while being the primary reason for hospitalization, may also be coded as other than most responsible diagnosis (ODx). Hence, if only MRDx-coded pneumonia cases are considered in analyses, cases in patients with COPD or other chronic conditions (where pneumonia would be coded as ODx) would not be included [10]. This may result in a misrepresentation (underestimate) of the actual burden of hospitalization for CAP. For this reason, understanding the contribution of ODx-coded cases is critical for a more comprehensive understanding of the hospitalization burden of CAP.

This study aimed to describe the burden of acute-care hospitalization for all-cause CAP in Canada, captured through administrative databases, and to assess the contribution of ODx-coded CAP cases to the overall disease burden.

## 2. Materials and Methods

### 2.1. Study Design and Data Source

This was an observational retrospective administrative database study. National-level hospitalization data were obtained from the Canadian Institute for Health Information (CIHI) Discharge Abstract Database (DAD), which contains demographic, administrative, and clinical data of patients admitted to hospitals in Canada (except Quebec), including patient age, the International Classification of Diseases Revision 10 (ICD-10-CA) code, date of hospital admission, intensive care unit (ICU) admission, length of hospital and ICU stay, and in-hospital death. The Canadian edition of ICD-10-CA codes was used to identify all-cause CAP cases hospitalized between 1 April 2009 and 31 March 2019 (Supplementary Table S1). CIHI’s Canadian Management Information System Database (CMDB) was used to estimate hospital stay costs. Population demographics were obtained from Statistics Canada.

### 2.2. Study Population

The study included cases in adults ≥ 50 years of age admitted for all-cause CAP across all provinces and territories in Canada (except Quebec). Cases were identified with ICD-10-CA codes J10-J18 recorded as type M or as pre-admit comorbidity type 1. Cases coded as type 2, denoting post-admit comorbidity, were excluded to focus on community-acquired pneumonia cases. A type M diagnosis is the diagnosis or condition that can be described as the most responsible for the patient’s stay in a hospital [11]. If more than one condition met this criterion, the condition held as most responsible for the greatest portion of the length of stay or greatest use of resources was selected. A type 1 diagnosis is a condition that existed prior to admission, has an associated ICD-10-CA code, and meets the requirements for a comorbidity [11]. CAP cases were classified to have CAP as MRDx (primary diagnosis) when CAP was captured as type M and ODx (secondary) when CAP was captured as type 1. To limit the inclusion of hospital-acquired pneumonia, and in accordance with the Canadian coding classification, subjects for whom pneumonia was classified as a post-admission comorbidity (type 2) were excluded. Additionally, cases where the same ICD-10-CA code of interest was captured in both type M and type 2 were also excluded. Patients who left the hospital against medical advice, before being seen/treated, or those who had missing demographic, cost, or missing/invalid health card information were excluded. The study focused on cases rather than patients—if the same patient had multiple pneumonia cases during the study period, each case would be counted separately.

### 2.3. Outcome Measures

The incidence rate, in-hospital mortality rate, hospital length of stay (LoS), hospitalization costs, ICU admission rates, and ICU length of stay for all-cause hospitalized CAP were assessed. Incidence rates were calculated as cases per 100,000 at-risk population. Denominator information was obtained from Statistics Canada’s annual population by age group and province. All in-hospital mortality rates were presented as deaths per 100 cases. The 95% confidence intervals (CIs) were calculated assuming a Poisson distribution. Hospital LoS was calculated as the median number of days in the hospital. To calculate the total average cost of each hospital stay, a top-down approach was used, in which a hospital-specific cost was assigned to each visit based on the resource intensity weight methodology from CMDB [12,13]. The estimates include the costs incurred by the hospital in providing services and exclude physician compensation since physicians are normally paid directly by the jurisdiction. The hospital costs include labour, nursing and allied health professionals, pharmacy (drugs), supplies, medical imaging, and laboratory, as well as indirect (overhead) costs. Due to changes in costing methodology between 2009–2013 and 2014–2018, comparison and trending of hospital LOS and costs are only reported for 2014–2018.

### 2.4. Stratification

Incidence and the in-hospital mortality rate were stratified by age group (50–64, 65–74, 75–84, and ≥85) or hospital case coding (MRDx and ODx). Hospital LOS and cost were stratified by age group, the presence or absence of ≥1 comorbidity, and case coding. For cases with an ICU stay, the length of ICU stay, hospital LoS, and costs of hospital stay were stratified by the presence or absence of ≥1 comorbidity, hospital case coding, and age group. Comorbidity at admission was defined as ≥1 if at least one ICD-10-CA code for any of the comorbidities of asthma, chronic respiratory disease, diabetes, cardiovascular disease, or obesity was captured (Supplementary Table S2).

### 2.5. Statistical Analysis

De-identified/anonymized aggregate-level data were provided by CIHI. Missing data were not imputed. To comply with CIHI’s Privacy and Confidentiality Policies, cell counts between 1 and 4 were suppressed and labelled as non-reportable, and where the risk of residual disclosure was apparent, double cell suppression was applied. In both the overall and stratified populations, all endpoints were analyzed descriptively, and no inferential analyses were conducted. For continuous variables, descriptive statistics such as count, mean, median, standard deviation, and interquartile range were provided as appropriate. For categorical variables, the number and proportion (%) of cases in each category were reported.

### 2.6. Ethics Approval

Ethics approval was not required as all data were anonymized and provided in an aggregate fashion. The personal health information collected by CIHI is governed by CIHI’s Privacy Policy, 2010.

## 3. Results

### 3.1. Incidence Rates of Hospitalization for All-Cause CAP

**Figure 1:**
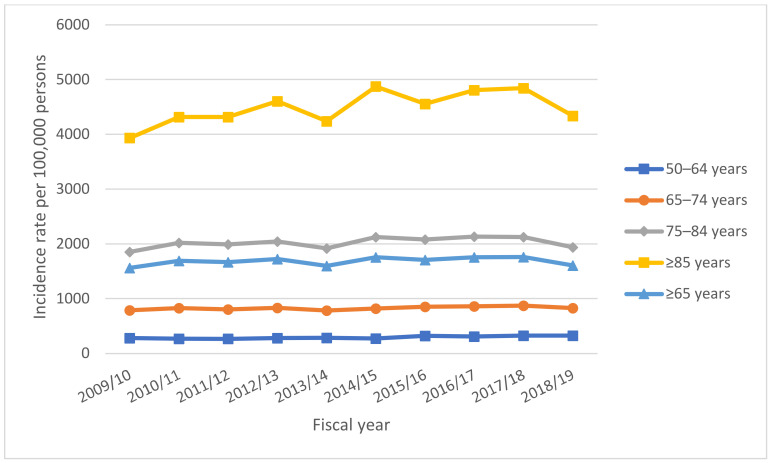
Incidence rates of acute-care hospitalization for all-cause community-acquired pneumonia, by age group, in fiscal years 2009–2018, Canada (excluding Quebec). Incidence rates were higher in older age groups compared to younger age groups.

Among adults aged ≥50 years, all-cause CAP acute-care hospitalization incidence rates increased from 805.66 per 100,000 in 2009–2010 to 896.94 per 100,000 in 2018–2019, representing an 11% increase (Table 1). Incidence rates increased in all age groups across the study period, and higher incidence rates were observed in older compared to younger age groups (Figure 1) (Supplementary Table S3). In 2018–2019, the incidence rate was the highest among the ≥85 age group (4337.20 per 100,000 adults), followed by the 75–84 (1936.27 per 100,000), 65–74 (826.83 per 100,000), and 50–64 age groups (326.02 per 100,000).

### 3.2. Incidence Rates Stratified by Case Coding

In 2018–2019, the incidence rate among adults ≥ 50 years was 361.80 and 535.14 per 100,000 population based on MRDx-coded and ODx-coded cases, respectively. Thus, MRDx-coded cases represented less than half of the total CAP incidence. Importantly, this trend was consistently observed over the ten-year study period, with more than half of hospitalized CAP cases in adults ≥ 50 years (range 57–60%) coded as ODx (Table 1). Similar trends were observed among adults ≥ 65 years, with ODx cases accounting for 60% of CAP cases in 2018–2019.

**Figure 2:**
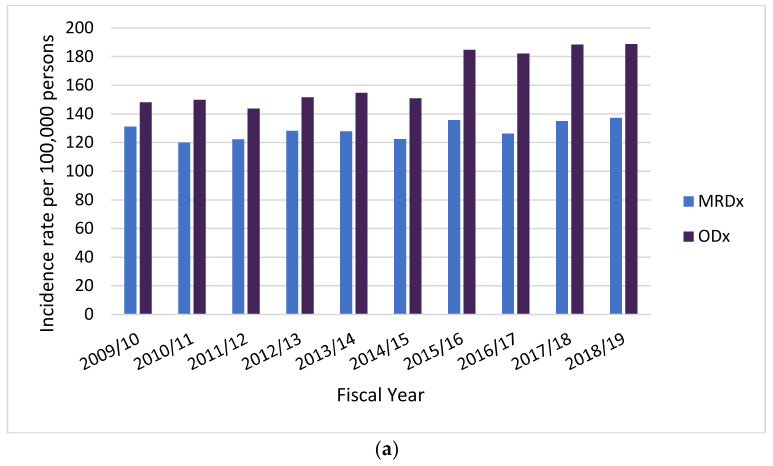

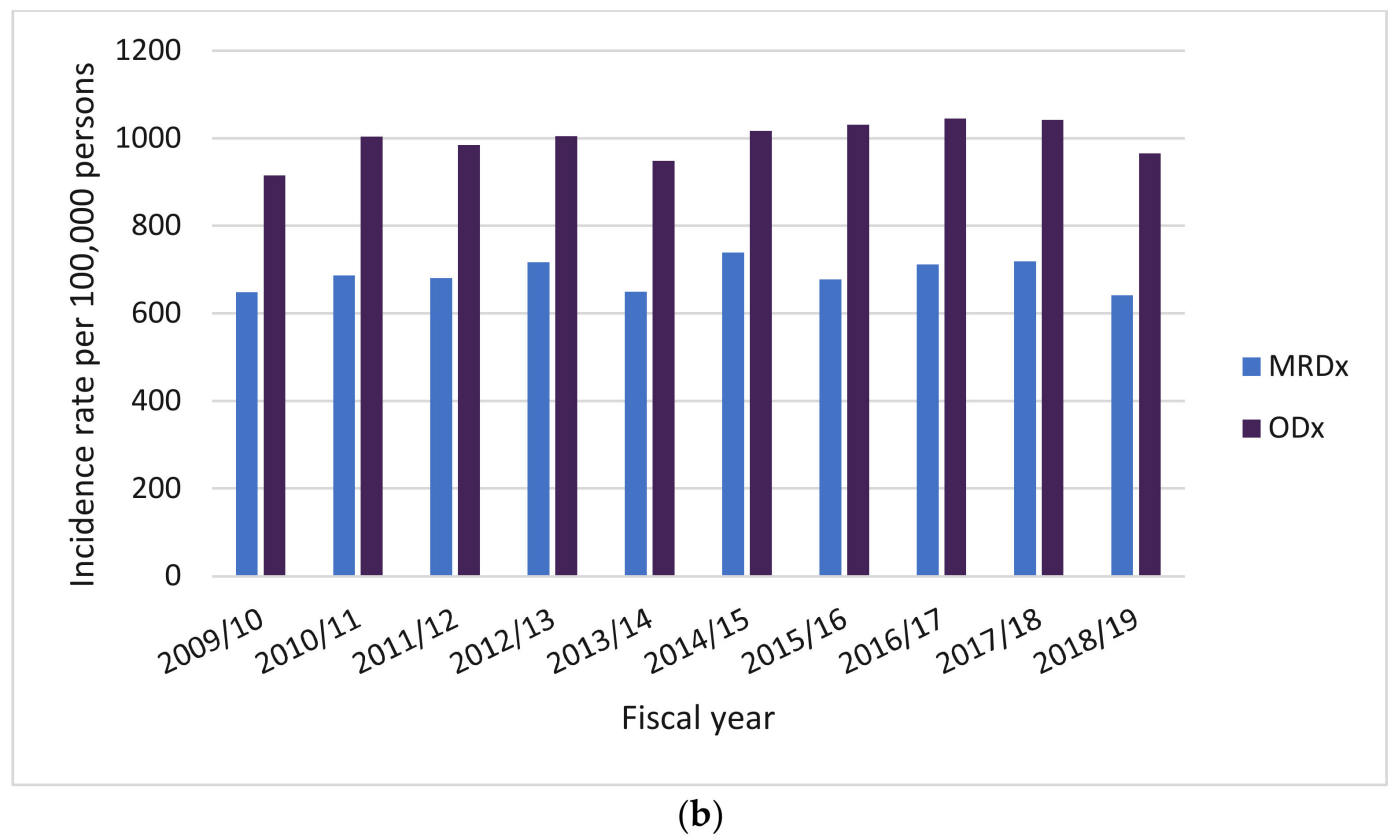
Incidence rates of acute-care hospitalization for all-cause community-acquired pneumonia, by case coding, in fiscal years 2009–2018. Incidence rates are presented for individuals (**a**) 50–64 years old and (**b**) ≥65 years old. Incidence rates estimated based on ODx-coded cases are higher than those estimated based on MRDx-coded cases across all age groups. Additional incidence rates per age group and case coding are available in Supplementary Table S4.

The trend of a higher incidence in older populations was also observed when stratifying by hospital case coding (Supplementary Table S4). However, incidence rates estimated based on ODx-coded cases are higher than those estimated based on MRDx-coded cases (Figure 2).

### 3.3. Hospitalized Cases with ICU Stay Stratified by Case Coding and Comorbidity

The proportion of cases requiring an ICU stay was higher when at least one comorbidity was present. This was true across study years and across age groups (Table 2). Among cases with ≥1 comorbidity of interest, a higher proportion of ODx-coded cases than MRDx-coded cases required an ICU stay across age groups and study years. In 2018–2019, among CAP cases with at least one comorbidity, 16.7% (1910/11,467) of MRDx-coded and 21.7% (9689/44,608) of ODx-coded cases required an ICU stay, while among cases without comorbidities of interest, the proportions were 4.8% (1299/27,292) and 12.2% (1546/12,720), respectively. In general, the number of CAP cases with an ICU stay increased from 2014–2015 to 2018–2019 across strata (Supplementary Table S5). The increase was higher among ODx-coded cases across age groups.

Generally, the median and total hospital LoS for ICU cases were longest for CAP cases coded as ODx that had ≥1 comorbidity of interest (ranging from 4 to 6 and from 11 to 12 days, respectively, in 2018–2019) and shortest for CAP coded as MRDx without comorbidity (ranging from 2 to 3 days and from 6 to 8 days, respectively, in 2018–2019) (Table 2).

The average total cost of hospitalization with a stay in intensive care followed a similar trend. In 2018–2019, costs were highest among ODx-coded CAP cases with comorbidity, ranging from CAD 26,073 to CAD 41,839 across age groups. Costs were lowest for the no-comorbidity and MRDx-coded groups, ranging from CAD 13,438 to CAD 22,082. The largest changes in the average total cost of hospital stay with ICU, between 2014–2015 and 2018–2019, occurred in the ≥85 age group, with a 32% increase (CAD 19,696–CAD 25,950) in costs for the comorbidity-present and MRDx groups, a 19% increase (CAD 21,902–CAD 26,074) for comorbidity-present and ODx groups, a 20% decrease (CAD 16,784–CAD 13,438) for comorbidity-absent and MRDx groups, and a 5% decrease in the comorbidity-absent and ODx stratification groups (Supplementary Table S5).

### 3.4. In-Hospital Mortality Rate for All-Cause CAP

Over the 10-year study period, the in-hospital mortality rate decreased between 2009–2010 and 2014–2015, then increased in 2015–2016, and remained relatively stable to 2018–2019 among adults aged ≥50 years (Table 3). Despite the slight uptick in 2015–2016, the fatality did not change much between 2009–2010 and 2018–2019, decreasing slightly from 14.47% to 13.10%. Like incidence rates, the in-hospital mortality for all-cause CAP increased with age, with the oldest age groups having the highest rates. In 2018–2019, the in-hospital mortality rate for age groups ≥85, 75–84, 65–74, and 50–64 was 17.82%, 14.06%, 10.64%, and 8.15% respectively. This trend was consistent across the study period (Supplementary Table S3).

### 3.5. In-Hospital Mortality Rate for All-Cause CAP Stratified by Case Coding

CAP cases coded as ODx had 1.65–1.87 times higher in-hospital mortality rates than CAP cases coded as MRDx (16.80% vs. 11.34% in 2009–2010 and 15.79% vs. 9.13% in 2018–2019) (Table 3). The overall in-hospital mortality rate for CAP cases coded as MRDx has declined over the ten-year period: from 11.34% in 2009–2010 to 9.13% in 2018–2019. While the mortality rate for ODx-coded cases also decreased over time, there was more fluctuation. In 2009–2010, the mortality rate was 16.80%, which decreased to 14.62% in 2013–2014, increased to 16.12% in 2016–2017, and then decreased to 15.79% in 2018–2019 (Table 3). Consistently across study years, the in-hospital mortality rate was at least twice as high for ODx-coded cases than MRDx-coded cases. The difference in the in-hospital mortality rate between MRDx- and ODx-coded cases increased over the study years.

### 3.6. Length of Stay and Total Cost of Hospitalization Stratified by Case Coding and Comorbidity

**Figure 3:**
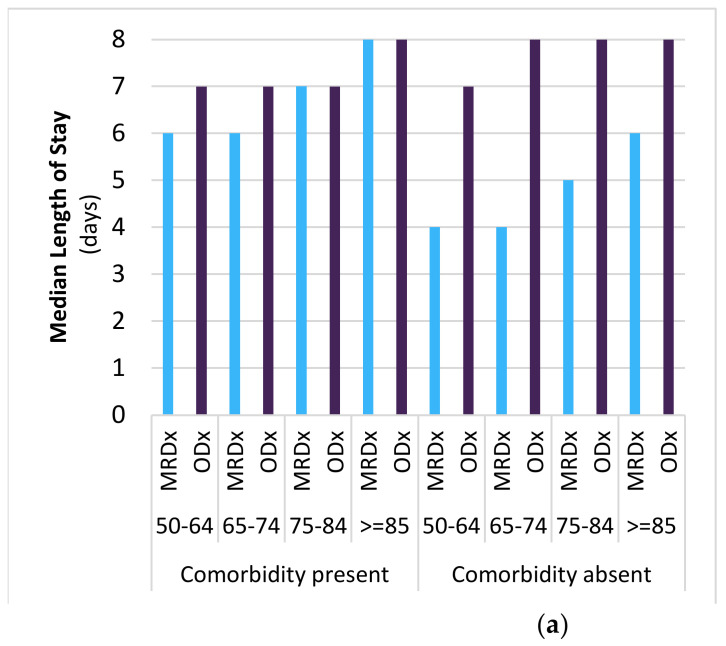

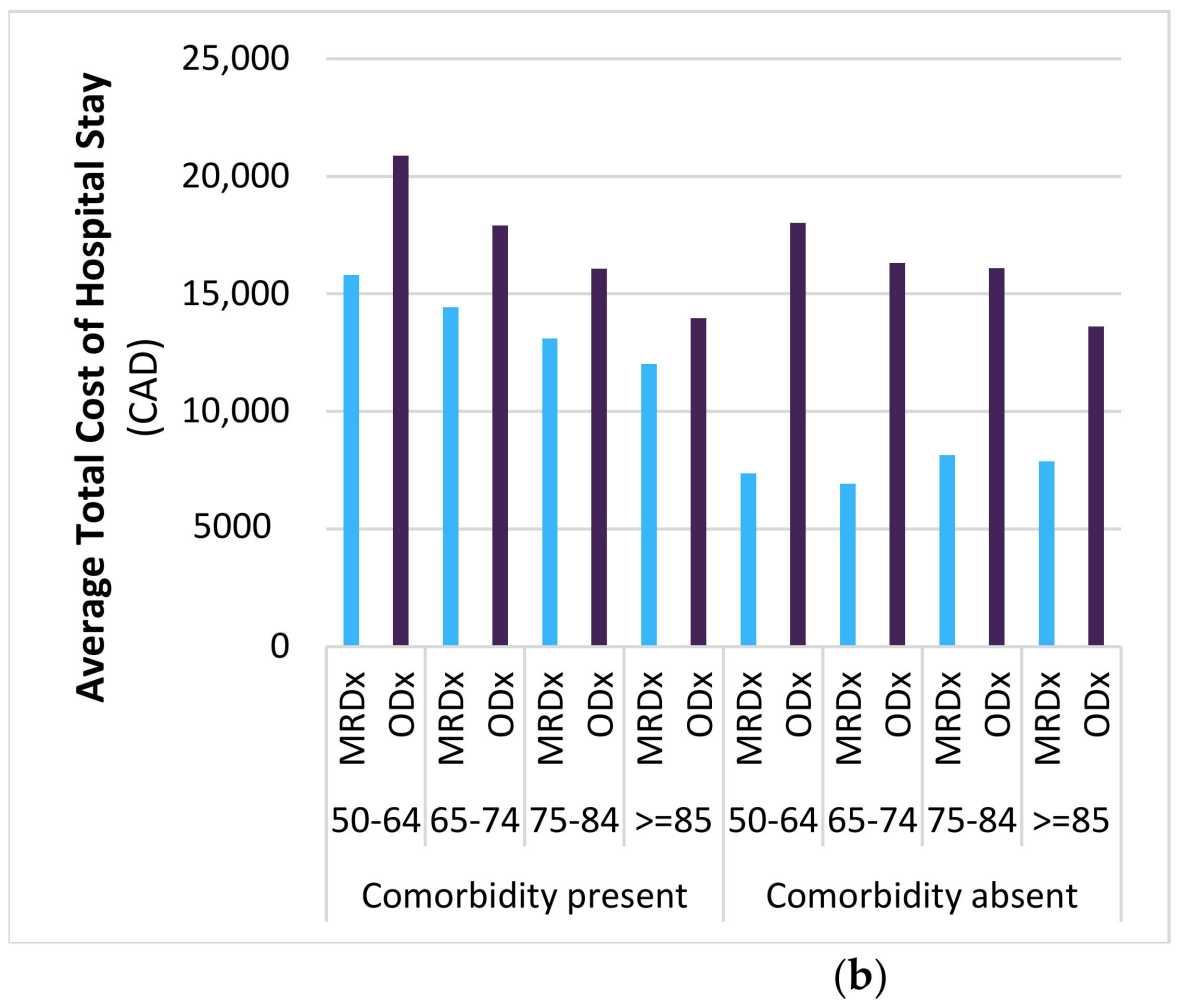
Length of stay and total cost of hospitalization for all-cause community-acquired pneumonia, by comorbidity, case coding, and age group, in fiscal year 2018, Canada (excluding Quebec). (**a**) The median hospital stay was generally longer for ODx-coded CAP cases, older age groups, and individuals with comorbidity present. (**b**) The average total cost of hospital stay was generally higher for ODx-coded CAP cases, younger age groups, and with comorbidity present. Additional data for fiscal year 2009–2018 are available in Supplementary Table S6.

Overall, among adults 50 years and older, the median LoS for all-cause CAP did not change notably between 2014–2015 (range across age groups: 4–10 days) and 2018–2019 (4–8 days) (Supplementary Table S6). The median LoS varied with age, with older adults having longer hospital stays than younger adults (Figure 3a). Conversely, the cost of hospitalization tended to be lower for the elderly.

Notably, across most age groups, the median hospital stay was longer for ODx-coded CAP cases. The median LoS ranged from 6 to 8 days among cases with at least one comorbidity and from 6 to 10 days among cases without any of the prespecified comorbidities. For MRDx-coded CAP cases, the median LoS ranged from 5 to 8 days when at least one comorbidity was present and from 3 to 6 days when there were no comorbidities. There were no large changes in the median LoS between 2014–2015 and 2018–2019 across comorbidity and hospital coding strata.

Correspondingly, the average total cost of hospital stay was the highest for CAP cases coded as ODx with at least one comorbidity (range CAD 13,964–CAD 20,876 in 2018–2019), while the average total cost of hospitalization was the lowest among CAP cases coded as MRDx and without comorbidities (range CAD 6940–CAD 7894 in 2018–2019) (Figure 3b). Interestingly, the average total cost of hospital stay decreased with increasing age for ODx cases with (CAD 20,876 and CAD 13,964 among 50–64 years and ≥85 years in 2018/19, respectively) or without (CAD 18,028 and CAD 13,608 among 50–64 and ≥85 years in 2018/19, respectively) comorbidity and for MRDx-coded cases with comorbidity (CAD 15,821 and CAD 12,040 among 50–64 and ≥85 years in 2018/19, respectively). However, costs tended to be similar across age groups or greater for older CAP cases coded as MRDx without comorbidity (CAD 7372 and CAD 7894 among 50–64 and ≥85 years in 2018/19, respectively).

Finally, the average cost of hospital stay increased between 2014–2015 and 2018–2019 across comorbidity and hospital coding strata.

## 4. Discussion

The public health burden of hospitalized CAP in Canada is substantial. In 2010, diseases of the respiratory system in Canada accounted for CAD 2.3 billion of inpatient hospital expenses [14]. This observational retrospective database analysis assessed the clinical and economic burden of acute-care hospitalization for all-cause CAP among adults aged ≥50 years in Canada. The analysis focused on the contribution of ODX-coded pneumonia cases to the overall disease burden during the 2010–2019 study period.

Over the 10-year study period, the incidence rate of all-cause CAP increased from 805.66 to 896.94 per 100,000 in adults aged ≥50 years. More than half of the annual all-cause CAP cases were coded as ODx. This suggests that estimates of the incidence rate based on analyses that focus solely on MRDx-coded cases would include less than half of the total all-cause CAP hospitalizations in Canada. Furthermore, ODx-coded CAP cases had a higher in-hospital mortality rate than CAP cases coded as MRDx, although the difference in mortality between ODx- and MRDx-coded cases decreased with age. The average cost of hospitalization was also higher for CAP cases coded as ODx that had at least one of the comorbidities of interest. There was a notable increase in the number of all-cause CAP hospitalizations requiring admission to an ICU in the last five years of the study, mostly in the ODx population. The ICU LoS was longer among cases coded as ODx that had at least one comorbidity, and the associated hospital stay costs were also higher for this population. The increase in hospital LoS, admission to ICU, and average cost in cases coded as ODx demonstrates that this population (likely represented by patients with other comorbidities) comprises a significant portion of all-cause pneumonia cases and likely requires more complex care at a higher cost than cases coded as MRDx.

While active population-based surveillance remains a gold standard in determining all-cause CAP incidence, its high cost means it is not always feasible, and administrative database analysis is often used to estimate the disease burden. At least four other database studies evaluated the disease burden of pneumonia in Canada. In an analysis conducted by the Conference Board of Canada (CBOC), two-thirds of pneumonia cases were coded as ODx. Furthermore, when compared to MRDx-coded cases, similar to our findings, ODx-coded cases had higher mortality, length of stay (LOS), and average cost per hospitalization [15,16]. Increasing hospital stays, mortality, and associated costs are likely related to comorbid conditions, as shown in our assessment. Adequate assessment of all-cause CAP is relevant when considering public health strategies among the adult population. A retrospective analysis conducted by McNeil et al. in 2016, also using CIHI data to evaluate trends in pneumonia incidence in Canada from 2004 to 2010 [5], reported similar results to our study. McNeil et al. noted that the incidence of pneumonia hospitalization was higher in older than younger populations, with a marked increase after 60 years of age. Morrow et al. used an administrative database from Quebec hospitals (Med-Echo) from 1991 to 2011 to determine the incidence of all-cause hospitalized pneumonia, using ICD-9 codes and considering only the primary diagnosis. The incidence in adults ≥65 years estimated by McNeil et al. (2016) was four times higher compared to that assessed by Morrow et al. (1537/100,000 vs. 333/100,000) [5,17]. Grenier et al. (2018), who also included published data from Quebec, reviewed data on all adults ≥65 years admitted at Centre intégré universitaire de santé et de services sociaux (CIUSSS) de l’Estrie in 2015 with a MRDx or ODx diagnosis of pneumonia. Among 243 included cases (radiological confirmation and without previous exposure to healthcare), 126 were identified with CAP as a principal diagnosis and 117 as a secondary diagnosis. In this particular study, pneumonia in COPD cases was more likely to be coded as a secondary diagnosis [10].

Several other studies have evaluated the impact of hospital coding on estimates of pneumonia incidence, mortality, and disease burden [18,19,20,21]. Smithee et al. [18] noted that a substantial number of pneumonia cases are being coded as COPD exacerbation, with pneumonia listed as the secondary diagnosis because coding practices for COPD require that the lower respiratory infection be sequenced after the COPD. Suaya et al. [20] also observed that hospitalized pneumonia cases were identified within the first six diagnostic fields at discharge, but only one-third of the cases were coded as the primary diagnosis. They concluded that the overall incidence of pneumonia hospitalizations is currently higher when considering not only the primary diagnosis for the analysis. In Germany, Theilacker et al. [21], by using the InGef database and considering hospitalized CAP cases as a primary or secondary CAP diagnosis, found that the incidence rate of hospitalized pneumonia increased by 1.5 times in those ≥ 60 years when they included pneumonia coded as both the primary and secondary diagnosis compared to only the primary diagnosis. These findings, as those of our study, suggest that estimates of all-cause CAP incidence relying only on the primary diagnosis when using administrative databases may underestimate the overall disease burden.

### Strengths and Limitations

The strengths of our study include the availability of 10-year period data, which allowed us to examine trends in the incidence of hospitalization rates over time. Administrative databases have proven to be useful when assessing clinical outcomes and costs associated with hospitalization and can provide information on all cases across Canada (except Quebec) stratified by age, case coding, and comorbidity, allowing for better estimation of the overall disease and economic burden of pneumonia [22]. The inclusion of both MRDx- and ODx-coded cases allowed us to better assess the clinical and economic burden of CAP and to demonstrate that ODx-coded CAP cases make up a majority of hospitalized all-cause CAP.

Although retrospective database analysis has been recognized as a useful tool for generating real-world evidence when evaluating healthcare decisions, there are some limitations to acknowledge. The data obtained have limited clinical information relating each ICD code to a pneumonia case; therefore, if one patient has had multiple hospitalizations due to pneumonia, each case would be counted separately, with the potential of overestimating the disease incidence. Length of hospital stay, mortality rates, and direct medical costs cannot be directly attributed to pneumonia over the other diagnoses that occurred with pneumonia. For these same reasons, case fatality rates cannot be estimated directly. While the completeness of incidence rate estimates is improved by the inclusion of ODx cases, the in-hospital mortality, hospital LOS, and total costs may be overestimated for CAP if comorbid conditions are more likely to result in death and/or a longer hospital stay (i.e., if death is due to the comorbid condition(s) rather than CAP). The degree to which these outcomes can be attributed to pneumonia varies. The attribution is likely to be high for COPD and congestive heart failure, which may have been caused by pneumonia infection, and therefore, pneumonia would be the primary driver of outcomes. While the comorbid condition(s) of ODx cases is likely heterogeneous and could be various diagnoses, this information was not captured as part of this study. Nevertheless, this study accurately reflects the burden of pneumonia hospitalizations that are further complicated by other comorbidities. Cost estimates are based on a case-level costing methodology, which represents hospital costs only and excludes costs related to physician compensation. Case costing values are estimates, and substitutes are employed for provinces and territories for which there is no estimated cost value attributed to an abstract. In the case of an estimated cost, specific costs such as medical compensation and termination benefits are excluded. Lastly, due to restricted availability, data from Quebec, which accounts for about a quarter of Canada’s population, were not included.

## 5. Conclusions

Our results suggest that excluding ODx-coded hospitalized CAP likely excludes the more complex pneumonia cases given that these cases had higher mortality, higher hospital costs, longer in-hospital stay, and were more likely to be admitted to the ICU. The incidence of CAP is higher, and this difference between MRDx- and ODx-coded cases is greater in older age groups (≥65 years). Therefore, studies focusing solely on MRDx-coded cases may underestimate both the clinical and economic burden of CAP, particularly in older individuals. These findings may generalize to other disease areas where burden is estimated using administrative hospitalization data with similar hospital coding practices, in which the exclusion of ODx-coded cases may lead to considerable underestimates of burden. This is an important finding with relevance for health policy and planning, specifically for assessing the need for and accurately assessing the potential clinical and budgetary impact of the current and future pneumococcal, and other, immunization programs.

## Figures and Tables

**Table 1:** Incidence rates of acute-care hospitalization for all-cause community-acquired pneumonia among adults ≥50 and ≥65 years of age, by case coding, in fiscal years 2009–2018, Canada (excluding Quebec).

			Total	MRDx	ODx
Fiscal Year	Number of Cases	Number in At-Risk Population	Incidence per 100,000 (95%CI)	Incidence per 100,000 (% of Total)	Incidence per 100,000 (% of Total)
≥50 years					
2009/10	68,758	8,534,355	805.66 (799.64, 811.68)	343.31 (42.61%)	462.35 (57.39%)
2010/11	74,743	8,796,371	849.70 (843.61, 855.79)	351.43 (41.36%)	498.27 (58.64%)
2011/12	75,859	9,059,910	837.30 (831.35, 843.26)	350.20 (41.82%)	487.10 (58.18%)
2012/13	81,656	9,323,656	875.79 (869.79, 881.80)	371.57 (42.43%)	504.22 (57.57%)
2013/14	79,837	9,604,233	831.27 (825.50, 837.04)	345.48 (41.56%)	485.79 (58.44%)
2014/15	88,512	9,871,173	896.67 (890.76, 902.58)	381.62 (42.56%)	515.06 (57.44%)
2015/16	91,939	10,106,612	909.69 (903.81, 915.57)	365.68 (40.20%)	544.01 (59.80%)
2016/17	96,165	10,322,705	931.59 (925.70, 937.48)	378.22 (40.60%)	553.36 (59.40%)
2017/18	100,211	10,522,292	952.37 (946.47, 958.27)	390.39 (40.99%)	561.98 (59.01%)
2018/19	96,087	10,712,807	896.94 (891.26, 902.61)	361.80 (40.34%)	535.14 (59.66%)
≥65 years					
2009/10	54,688	6,998,556	781.42 (774.87, 787.97)	648.36 (41%)	914.47 (59%)
2010/11	60,689	7,182,924	844.91 (838.18, 851.63)	686.77 (41%)	1003.05 (59%)
2011/12	61,594	7,398,020	832.57 (826.00, 839.15)	680.37 (41%)	225.97 (59%)
2012/13	66,354	7,706,800	860.98 (854.43, 867.53)	717.08 (42%)	1004.88 (58%)
2013/14	64,012	8,017,342	798.42 (792.23, 804.60)	649.12 (41%)	947.72 (59%)
2014/15	72,883	8,306,526	877.42 (871.05, 883.79)	738.46 (42%)	1016.38 (58%)
2015/16	73,298	8,585,416	853.75 (847.57, 859.93)	677.06 (40%)	1030.45 (60%)
2016/17	78,033	8,888,056	877.95 (871.79, 884.11)	711.45 (41%)	1044.46 (59%)
2017/18	81,079	9,214,198	879.94 (873.88, 885.99)	718.24 (41%)	1041.63 (59%)
2018/19	76,732	9,552,250	803.29 (797.60, 808.97)	640.88 (40%)	965.70 (60%)

Notes: MRDx: most responsible diagnosis; ODx: other than most responsible diagnosis.

**Table 2:** Intensive care unit length of stay and total cost of hospitalization for all-cause community-acquired pneumonia, by comorbidity, case coding, and age group, in fiscal year 2018–2019, Canada (excluding Quebec).

Comorbidity	Case Coding	Age Group	Number of Cases	Number of Cases with ICU Stay	Proportion of Cases with ICU Stay	Length of ICU Stay-Median (Days)	Length of Total Hospital Stay for ICU Cases—Median (Days)	Daily Cost of Hospital Stay for ICU Cases—Average (Standard Deviation)	Total Cost of Hospital Stay for ICU Cases—Average (Standard Deviation)
Present	MRDx (*N* = 11,467)	50–64	1857	529	28.5%	5	10	CAD 2431.22 (CAD 1858.09)	CAD 34,468.89 (CAD 42,293.78)
		65–74	2301	517	22.5%	5	10	CAD 2053.14 (CAD 1285.05)	CAD 29,482.20 (CAD 39,605.62)
		75–84	3180	514	16.2%	4	11	CAD 1904.32 (CAD 1250.60)	CAD 28,262.62 (CAD 40,550.58)
		≥85	4129	350	8.5%	4	11	CAD 1537.86 (CAD 1071.96)	CAD 25,949.64 (CAD 70,718.41)
	ODx (*N* = 44,608)	50–64	8023	2725	34.0%	5	11	CAD 2601.09 (CAD 2074.57)	CAD 41,839.16 (CAD 58,293.89)
		65–74	11,466	3061	26.7%	5	11	CAD 2258.20 (CAD 1489.29)	CAD 37,232.93 (CAD 67,820.33)
		75–84	13,717	2665	19.4%	4	11	CAD 2031.28 (CAD 1411.95)	CAD 34,639.84 (CAD 61,729.12)
		≥85	11,402	1238	10.9%	4	11	CAD 1741.29 (CAD 1383.45)	CAD 26,073.95 (CAD 42,085.72)
Absent	MRDx (*N* = 27,292)	50–64	6293	493	7.8%	3	7	CAD 1969.66 (CAD 1458.66)	CAD 22,082.92 (CAD 34,357.27)
		65–74	6053	360	5.9%	3	8	CAD 1586.58 (CAD 1113.19)	CAD 16,797.06 (CAD 25,930.36)
		75–84	7096	286	4.0%	3	8	CAD 1667.30 (CAD 1230.49)	CAD 17,362.88 (CAD 26,783.23)
		≥85	7850	160	2.0%	2	8	CAD 1474.23 (CAD 1430.95)	CAD 13,438.01 (CAD 15,393.63)
	ODx (*N* = 12,720)	50–64	3182	603	19.0%	4	11	CAD 2454.14 (CAD 1667.17)	CAD 34,902.43 (CAD 43,119.58)
		65–74	2935	437	14.9%	4	10	CAD 2220.14 (CAD 1675.02)	CAD 32,856.57 (CAD 50,708.57)
		75–84	3273	328	10.0%	3	10	CAD 1975.19 (CAD 1290.20)	CAD 27,692.54 (CAD 44,568.48)
		≥85	3330	178	5.3%	3	9	CAD 1856.61 (CAD 1745.90)	CAD 21,073.90 (CAD 23,532.19)

Notes: MRDx: most responsible diagnosis; ODx: other than most responsible diagnosis.

**Table 3:** In-hospital mortality rate for all-cause community-acquired pneumonia among adults ≥50 years of age, overall and by case coding, in fiscal years 2009–2018, Canada (excluding Quebec).

Fiscal Year	Number of Deaths	In-Hospital Mortality Rate-Deaths/100 Cases (95% CI)	Case Coding	Number of Deaths	In-Hospital Mortality Rate (Deaths/100 Cases)	In-Hospital Mortality Rate 95% CI
2009/2010	9952	14.47 (14.19, 14.76)	MRDx	3322	11.34	(10.95, 11.72)
			ODx	6630	16.80	(16.40, 17.21)
2010/2011	10,525	14.08 (13.81, 14.35)	MRDx	3476	11.24	(10.87, 11.62)
			ODx	7049	16.08	(15.71, 16.46)
2011/2012	10,216	13.47 (13.21, 13.73)	MRDx	3270	10.31	(9.95, 10.66)
			ODx	6946	15.74	(15.37, 16.11)
2012/2013	10,718	13.13 (12.88, 13.37)	MRDx	3477	10.04	(9.70, 10.37)
			ODx	7241	15.40	(15.05, 15.76)
2013/2014	10,064	12.61 (12.36, 12.85)	MRDx	3243	9.77	(9.44, 10.11)
			ODx	6821	14.62	(14.27, 14.97)
2014/2015	10,874	12.29 (12.05, 12.52)	MRDx	3519	9.34	(9.03, 9.65)
			ODx	7355	14.47	(14.14, 14.80)
2015/2016	12,184	13.25 (13.02, 13.49)	MRDx	3433	9.29	(8.98, 9.60)
			ODx	8751	15.92	(15.58, 16.25)
2016/2017	12,986	13.50 (13.27, 13.74)	MRDx	3776	9.67	(9.36, 9.98)
			ODx	9210	16.12	(15.79, 16.45)
2017/2018	13,129	13.10 (12.88, 13.33)	MRDx	3722	9.06	(8.77, 9.35)
			ODx	9407	15.91	(15.59, 16.23)
2018/2019	12,591	13.10 (12.87, 13.33)	MRDx	3539	9.13	(8.83, 9.43)
			ODx	9052	15.79	(15.46, 16.12)

Notes: MRDx: most responsible diagnosis; ODx: other than most responsible diagnosis.

## Data Availability

The data that support the findings of this study are available on request from the corresponding author. The data are not publicly available due to privacy or ethical restrictions.

## Supplementary Materials

The following supporting information can be downloaded at: https://www.mdpi.com/article/10.3390/vaccines11040748/s1, Table S1: ICD-10-CA diagnosis codes for all-cause CAP hospitalizations; Table S2: ICD-10-CA diagnosis codes for comorbidities of interest; Table S3: Incidence rates and in-hospital mortality of acute-care hospitalization for all-cause community-acquired pneumonia, by age group, in fiscal years 2009–2018, Canada (excluding Quebec); Table S4. Incidence rates of acute-care hospitalization and in-hospital mortality for all-cause community-acquired pneumonia, by case coding and age group, in fiscal years 2009–2018, Canada (excluding Quebec); Table S5. Intensive care unit length of stay and total cost of hospitalization for all-cause community-acquired pneumonia, by comorbidity, case coding, and age group, in fiscal years 2009–2018, Canada (excluding Quebec); Table S6. Length of stay and total cost of hospitalization for all-cause community-acquired pneumonia, by comorbidity, case coding, and age group, in fiscal years 2009–2018, Canada (excluding Quebec).
