# Supplementary material for: Burden of Acute-Care Hospitalization for Community-Acquired Pneumonia in Canadian Adults Aged 50 Years or Older: Focusing on Most Responsible Diagnosis Tells Only Part of the Story
Source: Vaccines (Basel). 2023 Mar 28;11(4):748. doi: 10.3390/vaccines11040748 (PMC10146146; doi:10.3390/vaccines11040748)
Supplement: Supplementary file 1 [file vaccines-11-00748-s001.zip › vaccines-2244664-supplementary.pdf]

Supplementary Table S1: ICD-10-CA diagnosis codes for all-cause CAP hospitalizations

ICD-10-CA Codes	ICD-10-CA Codes Description
**ALL-CAUSE PNEUMONIA (CAP)**	
J10.0	Influenza with pneumonia, seasonal influenza virus identified
J11.0	Influenza with pneumonia, virus not identified
J12	Viral pneumonia, not elsewhere classified
J12.0	Adenoviral pneumonia
J12.1	Respiratory syncytial virus pneumonia
J12.2	Parainfluenza virus pneumonia
J12.3	Human metapneumovirus pneumonia
J12.8	Other viral pneumonia
J12.9	Viral pneumonia, unspecified
J13	Pneumonia due to Streptococcus pneumoniae
J14	Pneumonia due to Haemophilus influenzae
J15	Bacterial pneumonia, not elsewhere classified
J15.0	Pneumonia due to Klebsiella pneumoniae
J15.1	Pneumonia due to Pseudomonas
J15.2	Pneumonia due to staphylococcus
J15.3	Pneumonia due to streptococcus, group B
J15.4	Pneumonia due to other streptococci
J15.5	Pneumonia due to Escherichia coli
J15.6	Pneumonia due to other Gram-negative bacteria
J15.7	Pneumonia due to Mycoplasma pneumoniae
J15.8	Other bacterial pneumonia
J15.9	Bacterial pneumonia, unspecified
J16	Pneumonia due to other infectious organisms, not elsewhere classified
J16.0	Chlamydial pneumonia
J16.8	Pneumonia due to other specified infectious organisms

J17	Pneumonia in diseases classified elsewhere
J17.0	Pneumonia in bacterial diseases classified elsewhere
J17.1	Pneumonia in viral diseases classified elsewhere
J17.2	Pneumonia in mycoses
J17.3	Pneumonia in parasitic diseases
J17.8	Pneumonia in other diseases classified elsewhere
J18	Pneumonia, organism unspecified
J18.0	Bronchopneumonia, unspecified
J18.1	Lobar pneumonia, unspecified
J18.2	Hypostatic pneumonia, unspecified
J18.8	Other pneumonia, organism unspecified
J18.9	Pneumonia, unspecified

Supplementary Table S2: ICD-10-CA diagnosis codes for comorbidities of interest

ICD-10-CA Codes	ICD-10-CA Codes Description
**J45**	Asthma
**J45.0**	Predominantly allergic asthma
**J45.00**	Predominantly allergic asthma without stated status asthmaticus
**J45.01**	Predominantly allergic asthma with stated status asthmaticus
**J45.1**	Nonallergic asthma
**J45.10**	Nonallergic asthma without stated status asthmaticus
**J45.11**	Nonallergic asthma with stated status asthmaticus
**J45.8**	Mixed asthma
**J45.80**	Mixed asthma without stated status asthmaticus
**J45.81**	Mixed asthma with stated status asthmaticus
**J45.9**	Asthma, unspecified
**J45.90**	Asthma, unspecified, without stated status asthmaticus
**J45.91**	Asthma, unspecified, with stated status asthmaticus

Chronic Respiratory Diseases (CRD)

ICD-10-CA Codes	ICD-10-CA Codes Description
**J40**	Bronchitis, not specified as acute or chronic
**J41**	Simple and mucopurulent chronic bronchitis
**J41.0**	Simple chronic bronchitis
**J41.1**	Mucopurulent chronic bronchitis
**J41.8**	Mixed simple and mucopurulent chronic bronchitis
**J42**	Unspecified chronic bronchitis
**J43**	Emphysema
**J43.0**	MacLeod's syndrome
**J43.1**	Panlobular emphysema
**J43.2**	Centrilobular emphysema
**J43.8**	Other emphysema

**J43.9**	Emphysema, unspecified
**J44**	Other chronic obstructive pulmonary disease
**J44.0**	Chronic obstructive pulmonary disease with acute lower respiratory infection
**J44.1**	Chronic obstructive pulmonary disease with acute exacerbation, unspecified
**J44.8**	Other specified chronic obstructive pulmonary disease
**J44.9**	Chronic obstructive pulmonary disease, unspecified
**J47**	Bronchiectasis
**J98**	Other respiratory disorders
**J98.0**	Diseases of bronchus, not elsewhere classified
**J98.2**	Interstitial emphysema
**J98.3**	Compensatory emphysema
**J98.4**	Other disorders of lung
**J98.8**	Other specified respiratory disorders
**J98.9**	Respiratory disorder, unspecified
**E84**	Cystic fibrosis
**E84.0**	Cystic fibrosis with pulmonary manifestations
**E84.1**	Cystic fibrosis with intestinal manifestations
**E84.8**	Cystic fibrosis with other manifestations
**E84.9**	Cystic fibrosis, unspecified

Diabetes

ICD-10-CA Codes	ICD-10-CA Codes Description
**E11**	Type 2 diabetes mellitus
**E11.0**	Type 2 diabetes mellitus with coma
**E11.1**	Type 2 diabetes mellitus with acidosis
**E11.10**	Type 2 diabetes mellitus with ketoacidosis
**E11.11**	Type 2 diabetes mellitus with lactic acidosis
**E11.12**	Type 2 diabetes mellitus with ketoacidosis with lactic acidosis
**E11.2**	Type 2 diabetes mellitus with kidney complications
**E11.20**	Type 2 diabetes mellitus with incipient diabetic nephropathy

**E11.23**	Type 2 diabetes mellitus with established or advanced kidney disease
**E11.28**	Type 2 diabetes mellitus with other specified kidney complication not elsewhere classified
**E11.3**	Type 2 diabetes mellitus with ophthalmic complications
**E11.30**	Type 2 diabetes mellitus with background retinopathy
**E11.31**	Type 2 diabetes mellitus with preproliferative retinopathy
**E11.32**	Type 2 diabetes mellitus with proliferative retinopathy
**E11.33**	Type 2 diabetes mellitus with other retinopathy
**E11.36**	Type 2 diabetes mellitus with advanced ophthalmic disease
**E11.38**	Type 2 diabetes mellitus with other specified ophthalmic complication not elsewhere classified
**E11.4**	Type 2 diabetes mellitus with neurological complications
**E11.40**	Type 2 diabetes mellitus with mononeuropathy
**E11.41**	Type 2 diabetes mellitus with polyneuropathy
**E11.42**	Type 2 diabetes mellitus with autonomic neuropathy
**E11.5**	Type 2 diabetes mellitus with circulatory complications
**E11.50**	Type 2 diabetes mellitus with peripheral angiopathy
**E11.51**	Type 2 diabetes mellitus with peripheral angiopathy with gangrene
**E11.52**	Type 2 diabetes mellitus with certain circulatory complications
**E11.6**	Type 2 diabetes mellitus with other specified complications
**E11.60**	Type 2 diabetes mellitus with musculoskeletal and connective tissue complication
**E11.61**	Type 2 diabetes mellitus with skin and subcutaneous tissue complication
**E11.62**	Type 2 diabetes mellitus with periodontal complication
**E11.63**	Type 2 diabetes mellitus with hypoglycaemia
**E11.64**	Type 2 diabetes mellitus with poor control, so described
**E11.68**	Type 2 diabetes mellitus with other specified complication, not elsewhere classified
**E11.7**	Type 2 diabetes mellitus with multiple complications
**E11.70**	Type 2 diabetes mellitus with foot ulcer (angiopathic)(neuropathic)
**E11.71**	Type 2 diabetes mellitus with foot ulcer (angiopathic) (neuropathic) with gangrene
**E11.78**	Type 2 diabetes mellitus with multiple other complications
**E11.9**	Type 2 diabetes mellitus without (mention of) complications

Cardiovascular Disease (CVD)

ICD-10-CA Codes	ICD-10-CA Codes Description
**I05**	Rheumatic mitral valve diseases
**I05.0**	Mitral stenosis
**I05.1**	Rheumatic mitral insufficiency
**I05.2**	Mitral stenosis with insufficiency
**I05.8**	Other mitral valve diseases
**I05.9**	Mitral valve disease, unspecified
**I06**	Rheumatic aortic valve diseases
**I06.0**	Rheumatic aortic stenosis
**I06.1**	Rheumatic aortic insufficiency
**I06.2**	Rheumatic aortic stenosis with insufficiency
**I06.8**	Other rheumatic aortic valve diseases
**I06.9**	Rheumatic aortic valve disease, unspecified
**I07**	Rheumatic tricuspid valve diseases
**I07.0**	Tricuspid stenosis
**I07.1**	Tricuspid insufficiency
**I07.2**	Tricuspid stenosis with insufficiency
**I07.8**	Other tricuspid valve diseases
**I07.9**	Tricuspid valve disease, unspecified
**I08**	Multiple valve diseases
**I08.0**	Disorders of both mitral and aortic valves
**I08.1**	Disorders of both mitral and tricuspid valves
**I08.2**	Disorders of both aortic and tricuspid valves
**I08.3**	Combined disorders of mitral, aortic and tricuspid valves
**I08.8**	Other multiple valve diseases
**I08.9**	Multiple valve disease, unspecified
**I09**	Other rheumatic heart diseases
**I09.0**	Rheumatic myocarditis
**I09.1**	Rheumatic diseases of endocardium, valve unspecified

**I09.2**	Chronic rheumatic pericarditis
**I09.8**	Other specified rheumatic heart diseases
**I09.9**	Rheumatic heart disease, unspecified
**I11**	Hypertensive heart disease
**I13**	Hypertensive heart and renal disease
**I20**	Angina pectoris
**I20.0**	Unstable angina
**I20.1**	Angina pectoris with documented spasm
**I20.8**	Other forms of angina pectoris
**I20.80**	Atypical angina
**I20.88**	Other forms of angina pectoris
**I20.9**	Angina pectoris, unspecified
**I21**	Acute myocardial infarction
**I21.0**	Acute transmural myocardial infarction of anterior wall
**I21.1**	Acute transmural myocardial infarction of inferior wall
**I21.2**	Acute transmural myocardial infarction of other sites
**I21.3**	Acute transmural myocardial infarction of unspecified site
**I21.4**	Acute subendocardial myocardial infarction
**I21.9**	Acute myocardial infarction, unspecified
**I24**	Other acute ischaemic heart diseases
**I24.0**	Coronary thrombosis not resulting in myocardial infarction
**I24.1**	Dressler's syndrome
**I24.8**	Other forms of acute ischaemic heart disease
**I24.9**	Acute ischaemic heart disease, unspecified
**I25**	Chronic ischaemic heart disease
**I25.0**	Atherosclerotic cardiovascular disease, so described
**I25.1**	Atherosclerotic heart disease
**I25.10**	Atherosclerotic heart disease of native coronary artery
**I25.11**	Atherosclerotic heart disease of autologous vein bypass graft
**I25.12**	Atherosclerotic heart disease of nonautologous biological bypass graft

**I25.13**	Atherosclerotic heart disease of artery bypass graft
**I25.14**	Atherosclerotic heart disease of unspecified type of bypass graft
**I25.15**	Atherosclerotic heart disease of coronary artery of transplanted heart
**I25.19**	Atherosclerotic heart disease of unspecified type of vessel, native or graft
**I25.2**	Old myocardial infarction
**I25.3**	Aneurysm of heart
**I25.4**	Coronary artery aneurysm and dissection
**I25.5**	Ischaemic cardiomyopathy
**I25.6**	Silent myocardial ischaemia
**I25.8**	Other forms of chronic ischaemic heart disease
**I25.9**	Chronic ischaemic heart disease, unspecified
**I27**	Other pulmonary heart diseases
**I27.0**	Primary pulmonary hypertension
**I27.1**	Kyphoscoliotic heart disease
**I27.2**	Other secondary pulmonary hypertension
**I27.8**	Other specified pulmonary heart diseases
**I27.9**	Pulmonary heart disease, unspecified
**I42**	Cardiomyopathy
**I42.0**	Dilated cardiomyopathy
**I42.1**	Obstructive hypertrophic cardiomyopathy
**I42.2**	Other hypertrophic cardiomyopathy
**I42.3**	Endomyocardial (eosinophilic) disease
**I42.4**	Endocardial fibroelastosis
**I42.5**	Other restrictive cardiomyopathy
**I42.6**	Alcoholic cardiomyopathy
**I42.7**	Cardiomyopathy due to drugs and other external agents
**I42.8**	Other cardiomyopathies
**I42.9**	Cardiomyopathy, unspecified
**I43**	Cardiomyopathy in diseases classified elsewhere
**I43.0**	Cardiomyopathy in infectious and parasitic diseases classified elsewhere
**I43.1**	Cardiomyopathy in metabolic diseases
**I43.2**	Cardiomyopathy in nutritional diseases
**I43.8**	Cardiomyopathy in other diseases classified elsewhere

**I44**	Atrioventricular and left bundle-branch block
**I44.0**	Atrioventricular block, first degree
**I44.1**	Atrioventricular block, second degree
**I44.2**	Atrioventricular block, complete
**I44.3**	Other and unspecified atrioventricular block
**I44.4**	Left anterior fascicular block
**I44.5**	Left posterior fascicular block
**I44.6**	Other and unspecified fascicular block
**I44.7**	Left bundle-branch block, unspecified
**I45**	Other conduction disorders
**I45.0**	Right fascicular block
**I45.1**	Other and unspecified right bundle-branch block
**I45.2**	Bifascicular block
**I45.3**	Trifascicular block
**I45.4**	Nonspecific intraventricular block
**I45.5**	Other specified heart block
**I45.6**	Pre-excitation syndrome
**I45.8**	Other specified conduction disorders
**I45.9**	Conduction disorder, unspecified
**I46**	Cardiac arrest
**I46.0**	Cardiac arrest with successful resuscitation
**I46.1**	Sudden cardiac death, so described
**I46.9**	Cardiac arrest, unspecified
**I47**	Paroxysmal tachycardia
**I47.0**	Re-entry ventricular arrhythmia
**I47.1**	Supraventricular tachycardia
**I47.2**	Ventricular tachycardia
**I47.9**	Paroxysmal tachycardia, unspecified
**I48**	Atrial fibrillation and flutter
**I48.0**	Atrial fibrillation
**I48.00**	Paroxysmal atrial fibrillation

**I48.01**	Persistent atrial fibrillation
**I48.02**	Chronic atrial fibrillation
**I48.1**	**Atrial flutter**
**I48.3**	Typical atrial flutter
**I48.4**	Atypical atrial flutter
**I48.9**	Atrial fibrillation and atrial flutter, unspecified
**I48.90**	Atrial fibrillation, unspecified
**I48.91**	Atrial flutter, unspecified
**I49**	Other cardiac arrhythmias
**I49.0**	Ventricular fibrillation and flutter
**I49.00**	Ventricular fibrillation
**I49.01**	Ventricular flutter
**I49.1**	Atrial premature depolarization
**I49.2**	Junctional premature depolarization
**I49.3**	Ventricular premature depolarization
**I49.4**	Other and unspecified premature depolarization
**I49.5**	Sick sinus syndrome
**I49.8**	Other specified cardiac arrhythmias
**I49.9**	Cardiac arrhythmia, unspecified
**I50**	Heart failure
**I50.0**	Congestive heart failure
**I50.1**	Left ventricular failure
**I50.9**	Heart failure, unspecified
**I51**	Complications and ill-defined descriptions of heart disease
**I51.0**	Cardiac septal defect, acquired
**I51.1**	Rupture of chordae tendineae, not elsewhere classified
**I51.2**	Rupture of papillary muscle, not elsewhere classified
**I51.3**	Intracardiac thrombosis, not elsewhere classified
**I51.4**	Myocarditis, unspecified
**I51.5**	Myocardial degeneration
**I51.6**	Cardiovascular disease, unspecified
**I51.7**	Cardiomegaly
**I51.8**	Other ill-defined heart diseases

**I51.9**	Heart disease, unspecified
**I52**	Other heart disorders in diseases classified elsewhere
**I52.0**	Other heart disorders in bacterial diseases classified elsewhere
**I52.1**	Other heart disorders in other infectious and parasitic diseases classified elsewhere
**I52.8**	Other heart disorders in other diseases classified elsewhere
**I63**	Cerebral infarction
**I63.0**	Cerebral infarction due to thrombosis of precerebral arteries
**I63.1**	Cerebral infarction due to embolism of precerebral arteries
**I63.2**	Cerebral infarction due to unspecified occlusion or stenosis of precerebral arteries
**I63.3**	Cerebral infarction due to thrombosis of cerebral arteries
**I63.4**	Cerebral infarction due to embolism of cerebral arteries
**I63.5**	Cerebral infarction due to unspecified occlusion or stenosis of cerebral arteries
**I63.6**	Cerebral infarction due to cerebral venous thrombosis, nonpyogenic
**I63.8**	Other cerebral infarction
**I63.9**	Cerebral infarction, unspecified
**J96**	Respiratory failure, not elsewhere classified
**J96.0**	Acute respiratory failure J96.00 J96.01 J96.09
**J96.00**	Acute respiratory failure, type 1 [hypoxic]
**J96.01**	Acute respiratory failure, type II [hypercapnic]
**J96.09**	Acute respiratory failure, type unspecified
**J96.1**	Chronic respiratory failure J96.10 J96.11 J96.19
**J96.10**	Chronic respiratory failure, type I [hypoxic]
**J96.11**	Chronic respiratory failure, type II [hypercapnic]
**J96.19**	Chronic respiratory failure, type unspecified
**J96.9**	Respiratory failure, unspecified J96.90 J96.91 J96.99
**J96.90**	Respiratory failure, unspecified, type I [hypoxic]
**J96.91**	Respiratory failure, unspecified, type II [hypercapnic]
**J96.99**	Respiratory failure, unspecified, type unspecified
**R09.2**	Respiratory arrest

Obesity

ICD-10-CA Codes	ICD-10-CA Codes Description
**Morbid Obesity**	
**E66.2**	Extreme obesity with alveolar hypoventilation
**Other Obesity**	
**E66**	Obesity
**E66.0**	Obesity due to excess calories
**E66.1**	Drug-induced obesity
**E66.8**	Other obesity
**E66.9**	Obesity, unspecified

Supplementary Table S3. Incidence rates and in-hospital mortality of acute-care hospitalization for all-cause community acquired pneumonia, by age group, fiscal year 2009-2018, Canada (excluding Quebec).

		Incidence			Fatality	
**Fiscal Year**	**Age group**	**Number of cases**	**Number in at-risk population**	**Incidence rate per 100,000 (95% CI)**	**Number of deaths**	**In-hospital mortality rate - deaths / 100 cases (95% CI)**
**2009/2010**	50-64	14,070	5,035,077	279.44 (274.82, 284.06)	1,031	7.33 (6.88, 7.77)
	65-74	14,628	1,854,677	788.71 (775.93, 801.49)	1,625	11.11 (10.57, 11.65)
	75-84	21,936	1,183,932	1852.81 (1828.29, 1877.33)	3,377	15.39 (14.88, 15.91)
	≥85	18,124	460,669	3934.28 (3877.00, 3991.56)	3,919	21.62 (20.95, 22.30)
	≥65	54,688	3,499,278	1562.84 (1549.74, 1575.94)	8,921	16.31 (15.97, 16.65)
**2010/2011**	50-64	14,054	5,204,909	270.01 (265.55, 274.48)	1,078	7.67 (7.21, 8.13)
	65-74	15,813	1,914,183	826.10 (813.22, 838.97)	1,668	10.55 (10.04, 11.05)
	75-84	24,159	1,197,309	2017.77 (1992.33, 2043.22)	3,532	14.62 (14.14, 15.10)
	≥85	20,717	479,970	4316.31 (4257.53, 4375.09)	4,247	20.50 (19.88, 21.12)
	≥65	60,689	3,591,462	1689.81 (1676.37, 1703.26)	9,447	15.57 (15.25, 15.88)
**2011/2012**	50-64	14,265	5,360,900	266.09 (261.73, 270.46)	949	6.65 (6.23, 7.08)
	65-74	16,012	1,989,308	804.90 (792.44, 817.37)	1,721	10.75 (10.24, 11.26)
	75-84	24,133	1,212,989	1989.55 (1964.45, 2014.65)	3,402	14.10 (13.62, 14.57)
	≥85	21,449	496,713	4318.19 (4260.40, 4375.98)	4,144	19.32 (18.73, 19.91)
	≥65	61,594	3,699,010	1665.15 (1652.00, 1678.30)	9,267	15.05 (14.74, 15.35)
**2012/2013**	50-64	15,302	5,470,256	279.73 (275.30, 284.16)	1,032	6.74 (6.33, 7.16)
	65-74	17,521	2,108,815	830.85 (818.54, 843.15)	1,742	9.94 (9.48, 10.41)
	75-84	25,100	1,229,256	2041.89 (2016.62, 2067.15)	3,403	13.56 (13.10, 14.01)
	≥85	23,733	515,329	4605.41 (4546.81, 4664.00)	4,541	19.13 (18.58, 19.69)
	≥65	66,354	3,853,400	1721.96 (1708.86, 1735.06)	9,686	14.60 (14.31, 14.89)
**2013/2014**	50-64	15,825	5,595,562	282.81 (278.41, 287.22)	1,043	6.59 (6.19, 6.99)
	65-74	17,460	2,227,126	783.97 (772.34, 795.60)	1,769	10.13 (9.66, 10.60)
	75-84	23,966	1,248,902	1918.97 (1894.67, 1943.26)	3,130	13.06 (12.60, 13.52)
	≥85	22,586	532,643	4240.36 (4185.06, 4295.67)	4,122	18.25 (17.69, 18.81)
	≥65	64,012	4,008,671	1596.84 (1584.47, 1609.21)	9,021	14.09 (13.80, 14.38)
**2014/2015**	50-64	15,629	5,717,910	273.33 (269.05, 277.62)	1,023	6.55 (6.14, 6.95)
	65-74	19,108	2,332,704	819.14 (807.52, 830.75)	1,873	9.80 (9.36, 10.25)
	75-84	27,014	1,271,749	2124.16 (2098.83, 2149.49)	3,292	12.19 (11.77, 12.60)
	≥85	26,761	548,810	4876.19 (4817.76, 4934.61)	4,686	17.51 (17.01, 18.01)
	≥65	72,883	4,153,263	1754.84 (1742.10, 1767.58)	9,851	13.52 (13.25, 13.78)
**2015/2016**	50-64	18,641	5,813,904	320.63 (316.03, 325.23)	1,505	8.07 (7.67, 8.48)
	65-74	20,772	2,437,632	852.14 (840.55, 863.73)	2,337	11.25 (10.79, 11.71)
	75-84	26,852	1,291,805	2078.64 (2053.78, 2103.50)	3,701	13.78 (13.34, 14.23)
	≥85	25,674	563,271	4558.02 (4502.26, 4613.77)	4,641	18.08 (17.56, 18.60)
	≥65	73,298	4,292,708	1707.50 (1695.14, 1719.86)	10,679	14.57 (14.29, 14.85)
**2016/2017**	50-64	18,132	5,878,677	308.44 (303.95, 312.93)	1,499	8.27 (7.85, 8.69)
	65-74	21,825	2,540,142	859.20 (847.80, 870.60)	2,413	11.06 (10.61, 11.50)
	75-84	28,171	1,320,512	2133.34 (2108.43, 2158.25)	3,934	13.96 (13.53, 14.40)
	≥85	28,037	583,374	4806.01 (4749.75, 4862.26)	5,140	18.33 (17.83, 18.83)

	≥65	78,033	4,444,028	1755.91 (1743.59, 1768.23)	11,487	14.72 (14.45, 14.99)
**2017/2018**	50-64	19,132	5,915,193	323.44 (318.86, 328.02)	1,479	7.73 (7.34, 8.12)
	65-74	23,099	2,647,753	872.40 (861.15, 883.65)	2,505	10.84 (10.42, 11.27)
	75-84	28,897	1,359,020	2126.31 (2101.80, 2150.83)	3,892	13.47 (13.05, 13.89)
	≥85	29,083	600,326	4844.53 (4788.86, 4900.21)	5,253	18.06 (17.57, 18.55)
	≥65	81,079	4,607,099	1759.87 (1747.76, 1771.98)	11,650	14.37 (14.11, 14.63)
**2018/2019**	50-64	19,355	5,936,682	326.02 (321.43, 330.62)	1,577	8.15 (7.75, 8.55)
	65-74	22,755	2,752,092	826.83 (816.08, 837.57)	2,421	10.64 (10.22, 11.06)
	75-84	27,266	1,408,175	1936.27 (1913.28, 1959.25)	3,833	14.06 (13.61, 14.50)
	≥85	26,711	615,858	4337.20 (4285.19, 4389.22)	4,760	17.82 (17.31, 18.33)
	≥65	76,732	4,776,125	1606.57 (1595.21, 1617.94)	11,014	14.35 (14.09, 14.62)

Supplementary Table S4. Incidence rates of acute-care hospitalization and in-hospital mortality for all-cause community acquired pneumonia, by case coding and age group, fiscal year 2009-2018, Canada (excluding Quebec).

Fiscal Year	Case coding	Age group	Number of cases	Number in at-risk population	Incidence rate per 100,000	Incidence rate per 100,000 (95% CI)	Number of deaths	In-hospital mortality rate (deaths / 100 cases)	In-hospital mortality rate 95% CI
2009/10	MRDx	50-64	6,611	5,035,077	131.30	(128.13, 134.46)	278	4.21	(3.71, 4.70)
		65-74	5,581	1,854,677	300.91	(293.02, 308.81)	419	7.51	(6.79, 8.23)
		75-84	8,732	1,183,932	737.54	(722.07, 753.01)	1,020	11.68	(10.96, 12.40)
		≥85	8,375	460,669	1818.01	(1779.07, 1856.94)	1,605	19.16	(18.23, 20.10)
		≥65	22,688	3,499,278	648.36	(639.93, 656.80)	3,044	13.42	(12.94, 13.89)
	ODx	50-64	7,459	5,035,077	148.14	(144.78, 151.50)	753	10.10	(9.37, 10.82)
		65-74	9,047	1,854,677	487.79	(477.74, 497.85)	1,206	13.33	(12.58, 14.08)
		75-84	13,204	1,183,932	1115.27	(1096.24, 1134.29)	2,357	17.85	(17.13, 18.57)
		≥85	9,749	460,669	2116.27	(2074.26, 2158.28)	2,314	23.74	(22.77, 24.70)
		≥65	32,000	3,499,278	914.47	(904.45, 924.49)	5,877	18.37	(17.90, 18.84)
2010/11	MRDx	50-64	6,248	5,204,909	120.04	(117.06, 123.02)	293	4.69	(4.15, 5.23)
		65-74	5,901	1,914,183	308.28	(300.41, 316.14)	421	7.13	(6.45, 7.82)
		75-84	9,381	1,197,309	783.51	(767.65, 799.36)	1,029	10.97	(10.30, 11.64)
		≥85	9,383	479,970	1954.91	(1915.36, 1994.47)	1,733	18.47	(17.60, 19.34)
		≥65	24,665	3,591,462	686.77	(678.20, 695.34)	3,183	12.90	(12.46, 13.35)
	ODx	50-64	7,806	5,204,909	149.97	(146.65, 153.30)	785	10.06	(9.35, 10.76)
		65-74	9,912	1,914,183	517.82	(507.62, 528.01)	1,247	12.58	(11.88, 13.28)
		75-84	14,778	1,197,309	1234.27	(1214.37, 1254.17)	2,503	16.94	(16.27, 17.60)
		≥85	11,334	479,970	2361.40	(2317.92, 2404.87)	2,514	22.18	(21.31, 23.05)
		≥65	36,024	3,591,462	1003.05	(992.69, 1013.40)	6,264	17.39	(16.96, 17.82)
2011/12	MRDx	50-64	6,561	5,360,900	122.39	(119.42, 125.35)	255	3.89	(3.41, 4.36)
		65-74	6,134	1,989,308	308.35	(300.63, 316.07)	420	6.85	(6.19, 7.50)
		75-84	9,264	1,212,989	763.73	(748.18, 779.29)	948	10.23	(9.58, 10.88)
		≥85	9,769	496,713	1966.73	(1927.73, 2005.73)	1,647	16.86	(16.05, 17.67)
		≥65	25,167	3,699,010	680.37	(671.97, 688.78)	3,015	11.98	(11.55, 12.41)
	ODx	50-64	7,704	5,360,900	143.71	(140.50, 146.92)	694	9.01	(8.34, 9.68)
		65-74	9,878	1,989,308	496.55	(486.76, 506.35)	1,301	13.17	(12.45, 13.89)
		75-84	14,869	1,212,989	1225.81	(1206.11, 1245.52)	2,454	16.50	(15.85, 17.16)
		≥85	11,680	496,713	2351.46	(2308.81, 2394.10)	2,497	21.38	(20.54, 22.22)
		≥65	36,427	3,699,010	984.78	(974.66, 994.89)	6,252	17.16	(16.74, 17.59)
2012/13	MRDx	50-64	7,012	5,470,256	128.18	(125.18, 131.18)	311	4.44	(3.94, 4.93)
		65-74	6,677	2,108,815	316.62	(309.03, 324.22)	423	6.34	(5.73, 6.94)
		75-84	9,930	1,229,256	807.81	(791.92, 823.69)	973	9.80	(9.18, 10.41)
		≥85	11,025	515,329	2139.41	(2099.47, 2179.35)	1,770	16.05	(15.31, 16.80)
		≥65	27,632	3,853,400	717.08	(708.63, 725.54)	3,166	11.46	(11.06, 11.86)
	ODx	50-64	8,290	5,470,256	151.55	(148.28, 154.81)	721	8.70	(8.06, 9.33)
		65-74	10,844	2,108,815	514.22	(504.54, 523.90)	1,319	12.16	(11.51, 12.82)
		75-84	15,170	1,229,256	1234.08	(1214.44, 1253.72)	2,430	16.02	(15.38, 16.66)
		≥85	12,708	515,329	2466.00	(2423.12, 2508.87)	2,771	21.81	(20.99, 22.62)
		≥65	38,722	3,853,400	1004.88	(994.87, 1014.89)	6,520	16.84	(16.43, 17.25)
2013/14	MRDx	50-64	7,160	5,595,562	127.96	(124.99, 130.92)	292	4.08	(3.61, 4.55)
		65-74	6,541	2,227,126	293.70	(286.58, 300.81)	415	6.34	(5.73, 6.96)
		75-84	9,178	1,248,902	734.89	(719.85, 749.92)	909	9.90	(9.26, 10.55)
		≥85	10,302	532,643	1934.13	(1896.78, 1971.48)	1,627	15.79	(15.03, 16.56)
		≥65	26,021	4,008,671	649.12	(641.23, 657.00)	2,951	11.34	(10.93, 11.75)
	ODx	50-64	8,665	5,595,562	154.85	(151.59, 158.12)	751	8.67	(8.05, 9.29)

		65-74	10,919	2,227,126	490.27	(481.08, 499.47)	1,354	12.40	(11.74, 13.06)
		75-84	14,788	1,248,902	1184.08	(1165.00, 1203.16)	2,221	15.02	(14.39, 15.64)
		≥85	12,284	532,643	2306.24	(2265.45, 2347.02)	2,495	20.31	(19.51, 21.11)
		≥65	37,991	4,008,671	947.72	(938.19, 957.25)	6,070	15.98	(15.58, 16.38)
2014/15	MRDx	50-64	7,000	5,717,910	122.42	(119.55, 125.29)	294	4.20	(3.72, 4.68)
		65-74	7,299	2,332,704	312.90	(305.72, 320.08)	446	6.11	(5.54, 6.68)
		75-84	10,675	1,271,749	839.40	(823.47, 855.32)	934	8.75	(8.19, 9.31)
		≥85	12,696	548,810	2313.37	(2273.13, 2353.61)	1,845	14.53	(13.87, 15.20)
		≥65	30,670	4,153,263	738.46	(730.19, 746.72)	3,225	10.52	(10.15, 10.88)
	ODx	50-64	86,29	5,717,910	150.91	(147.73, 154.10)	729	8.45	(7.83, 9.06)
		65-74	11,809	2,332,704	506.24	(497.11, 515.37)	1,427	12.08	(11.46, 12.71)
		75-84	16,339	1,271,749	1284.77	(1265.07, 1304.47)	2,358	14.43	(13.85, 15.01)
		≥85	14,065	548,810	2562.82	(2520.46, 2605.17)	2,841	20.20	(19.46, 20.94)
		≥65	42,213	4,153,263	1016.38	(1006.69, 1026.08)	6,626	15.70	(15.32, 16.07)
2015/16	MRDx	50-64	7,894	5,813,904	135.78	(132.78, 138.77)	309	3.91	(3.48, 4.35)
		65-74	7,501	2,437,632	307.72	(300.75, 314.68)	491	6.55	(5.97, 7.12)
		75-84	10,183	1,291,805	788.28	(772.97, 803.59)	953	9.36	(8.76, 9.95)
		≥85	11,380	563,271	2020.34	(1983.22, 2057.46)	1,680	14.76	(14.06, 15.47)
		≥65	29,064	4,292,708	677.06	(669.27, 684.84)	3,124	10.75	(10.37, 11.13)
	ODx	50-64	10,747	5,813,904	184.85	(181.36, 188.34)	1,196	11.13	(10.50, 11.76)
		65-74	13,271	2,437,632	544.42	(535.16, 553.68)	1,846	13.91	(13.28, 14.54)
		75-84	16,669	1,291,805	1290.37	(1270.78, 1309.95)	2,748	16.49	(15.87, 17.10)
		≥85	14,294	563,271	2537.68	(2496.08, 2579.28)	2,961	20.71	(19.97, 21.46)
		≥65	44,234	4,292,708	1030.45	(1020.84, 1040.05)	7,555	17.08	(16.69, 17.46)
2016/17	MRDx	50-64	7,426	5,878,677	126.32	(123.45, 129.19)	314	4.23	(3.76, 4.70)
		65-74	8,059	2,540,142	317.27	(310.34, 324.19)	519	6.44	(5.89, 6.99)
		75-84	10,731	1,320,512	812.64	(797.26, 828.02)	1,063	9.91	(9.31, 10.50)
		≥85	12,827	583,374	2198.76	(2160.71, 2236.81)	1,880	14.66	(13.99, 15.32)
		≥65	31,617	4,444,028	711.45	(703.61, 719.29)	3,462	10.95	(10.59, 11.31)
	ODx	50-64	10,706	5,878,677	182.12	(178.67, 185.57)	1,185	11.07	(10.44, 11.70)
		65-74	13,766	2,540,142	541.94	(532.89, 550.99)	1,894	13.76	(13.14, 14.38)
		75-84	17,440	1,320,512	1320.70	(1301.10, 1340.30)	2,871	16.46	(15.86, 17.06)
		≥85	15,210	583,374	2607.25	(2565.81, 2648.68)	3,260	21.43	(20.70, 22.17)
		≥65	46,416	4,444,028	1044.46	(1034.96, 1053.96)	8,025	17.29	(16.91, 17.67)
2017/18	MRDx	50-64	7,988	5,915,193	135.04	(132.08, 138.00)	307	3.84	(3.41, 4.27)
		65-74	8,519	2,647,753	321.74	(314.91, 328.58)	527	6.19	(5.66, 6.71)
		75-84	11,197	1,359,020	823.90	(808.64, 839.16)	1,008	9.00	(8.45, 9.56)
		≥85	13,374	600,326	2227.79	(2190.03, 2265.55)	1,880	14.06	(13.42, 14.69)
		≥65	33,090	4,607,099	718.24	(710.50, 725.98)	3,415	10.32	(9.97, 10.67)
	ODx	50-64	11,144	5,915,193	188.40	(184.90, 191.89)	1,172	10.52	(9.91, 11.12)
		65-74	14,580	2,647,753	550.66	(541.72, 559.59)	1,978	13.57	(12.97, 14.16)
		75-84	17,700	1,359,020	1302.41	(1283.22, 1321.60)	2,884	16.29	(15.70, 16.89)
		≥85	15,709	600,326	2616.74	(2575.82, 2657.67)	3,373	21.47	(20.75, 22.20)
		≥65	47,989	4,607,099	1041.63	(1032.31, 1050.95)	8,235	17.16	(16.79, 17.53)
2018/19	MRDx	50-64	8,150	5,936,682	137.28	(134.30, 140.26)	343	4.21	(3.76, 4.65)
		65-74	8,354	2,752,092	303.55	(297.04, 310.06)	518	6.20	(5.67, 6.73)
		75-84	10,276	1,408,175	729.74	(715.63, 743.85)	970	9.44	(8.85, 10.03)
		≥85	11,979	615,858	1945.09	(1910.26, 1979.92)	1,708	14.26	(13.58, 14.93)
		≥65	30,609	4,776,125	640.88	(633.70, 648.05)	3,196	10.44	(10.08, 10.80)
	ODx	50-64	11,205	5,936,682	188.74	(185.25, 192.24)	1,234	11.01	(10.40, 11.63)
		65-74	14,401	2,752,092	523.27	(514.73, 531.82)	1,903	13.21	(12.62, 13.81)
		75-84	16,990	1,408,175	1206.53	(1188.38, 1224.67)	2,863	16.85	(16.23, 17.47)

		≥85	14,732	615,858	2392.11	(2353.48, 2430.74)	3,052	20.72	(19.98, 21.45)
		≥65	46,123	4,776,125	965.70	(956.89, 974.51)	7818	16.95	(16.57, 17.33)

Notes: MRDx: most responsible diagnosis; ODx: other than most responsible diagnosis

Supplementary Table S5. Intensive care unit length of stay and total cost of hospitalization for all-cause community acquired pneumonia, by comorbidity, case coding and age group, fiscal year 2009-2018, Canada (excluding Quebec).

Fiscal Year	Comorbidity	Case coding	Age group	Number of cases	Number of cases with ICU stay	Proportion of cases with ICU stay	Length of ICU stay - median (days)	Length of total hospital stay for ICU cases - median (days)	Daily cost of hospital stay for ICU cases - average (standard deviation)	Total cost of hospital stay for ICU cases - average (standard deviation)
2014/2015	Present	MRDx (N=10,609)	50-64	1,506	360	23.9%	5	10	$2,193.14 ($1,476.29)	$31,466.74 ($38,942.09)
			65-74	1,920	407	21.2%	5	11	$1,944.13 ($1,348.61)	$29,214.44 ($36,195.67)
			75-84	3,165	468	14.8%	4	11	$1,735.02 ($1,113.34)	$25,552.19 ($45,989.67)
			≥85	4,018	304	7.6%	4	10	$1,589.37 ($1,089.99)	$19,696.40 ($26,585.79)
		ODx (N=40,637)	50-64	6,227	1,718	27.6%	5	11	$2,387.16 ($1,728.40)	$39,932.23 ($78,951.61)
			65-74	9,540	2,168	22.7%	5	11	$2,070.34 ($1,468.20)	$31,915.99 ($46,617.99)
			75-84	13,663	2,247	16.4%	5	11	$1,874.34 ($1,395.01)	$28,977.59 ($47,884.02)
			≥85	11,207	1,017	9.1%	4	11	$1,570.08 ($1,161.68)	$21,902.10 ($30,951.02)
	Absent	MRDx (N=27,061)	50-64	5,494	400	7.3%	4	8	$1,698.86 ($1,280.57)	$19,896.37 ($32,543.96)
			65-74	5,379	320	5.9%	3	8	$1,574.91 ($1,395.22)	$19,497.25 ($36,074.89)
			75-84	7,510	298	4.0%	3	7	$1,570.34 ($1,562.73)	$15,636.72 ($20,212.67)
			≥85	8,678	154	1.8%	3	9	$1,313.28 ($1,076.71)	$16,784.09 ($36,085.88)
		ODx (N=10,205)	50-64	2,402	422	17.6%	4	11	$2,186.14 ($1,474.88)	$34,056.84 ($43,996.80)
			65-74	2,269	276	12.2%	4	11	$2,026.85 ($1,359.82)	$32,229.76 ($43,503.10)
			75-84	2,676	212	7.9%	3	11	$1,834.73 ($1,210.33)	$27,022.48 ($31,930.01)
			≥85	2,858	94	3.3%	3	11	$1,751.30 ($1,173.50)	$22,267.41 ($23,307.27)
2015/2016	Present	MRDx (N=10,553)	50-64	1,736	500	28.8%	5	10	$2,324.22 ($1,541.10)	$32,097.11 ($37,160.94)
			65-74	2,013	444	22.1%	4	10	$2,168.24 ($1,657.00)	$28,806.38 ($42,438.00)
			75-84	3,120	491	15.7%	4	10	$1,997.37 ($1,334.51)	$26,041.89 ($31,421.03)
			≥85	3,684	302	8.2%	3	10	$1,526.06 ($1,176.48)	$23,394.96 ($39,815.17)
		ODx (N=43,075)	50-64	7,615	2,465	32.4%	5	11	$2,524.78 ($1,827.90)	$40,781.91 ($57,825.43)
			65-74	10,608	2,918	27.5%	5	11	$2,222.86 ($1,664.06)	$37,320.95 ($66,136.46)
			75-84	13,601	2,551	18.8%	5	11	$1,963.85 ($1,450.33)	$31,733.49 ($46,315.14)
			≥85	11,251	1,209	10.7%	4	11	$1,622.34 ($1,175.03)	$24,094.71 ($49,148.07)
	Absent	MRDx (N=26,405)	50-64	6,158	498	8.1%	3	7	$1,769.38 ($1,219.46)	$18,739.74 ($28,786.72)
			65-74	5,488	346	6.3%	3	7	$1,649.48 ($1,156.77)	$17,095.78 ($30,835.93)
			75-84	7,063	298	4.2%	3	7	$1,426.65 ($939.77)	$15,002.94 ($27,995.91)

			≥85	7,696	150	1.9%	2	7	$1,519.57 ($970.33)	$13,095.09 ($15,065.09)
		ODx (N=11,906)	50-64	3,132	666	21.3%	4	10	$2,331.38 ($1,618.37)	$38,125.41 ($57,119.51)
			65-74	2,663	377	14.2%	4	11	$2,059.97 ($1,266.59)	$35,952.64 ($53,698.69)
			75-84	3,068	305	9.9%	3	11	$1,802.18 ($1,241.49)	$29,639.41 ($53,077.46)
			≥85	3,043	130	4.3%	2	9	$1,525.53 ($1,054.10)	$21,183.66 ($26,569.24)
2016/2017	Present	MRDx (N=11,328)	50-64	1,688	521	30.9%	5	10	$2,324.45 ($1,618.14)	$31,316.81 ($40,169.18)
			65-74	2,153	495	23.0%	4	10	$2,152.13 ($1,495.13)	$29,278.91 ($36,230.24)
			75-84	3,261	502	15.4%	4	10	$1,895.39 ($1,236.85)	$28,618.01 ($39,871.44)
			≥85	4,226	337	8.0%	4	10	$1,607.63 ($1,117.57)	$19,055.35 ($21,499.66)
		ODx (N=44,642)	50-64	7,604	2,533	33.3%	5	11	$2,451.86 ($1,737.21)	$39,968.82 ($59,825.52)
			65-74	10,959	2,967	27.1%	5	11	$2,219.33 ($1,487.97)	$37,397.91 ($60,113.64)
			75-84	14,189	2,649	18.7%	4	11	$1,942.00 ($1,354.10)	$31,280.91 ($58,205.01)
			≥85	11,890	1,239	10.4%	4	10	$1,686.06 ($1,483.89)	$23,422.93 ($40,147.14)
	Absent	MRDx (N=27,715)	50-64	5,738	442	7.7%	3	7	$1,773.48 ($1,427.84)	$18,308.91 ($25,619.91)
			65-74	5,906	340	5.8%	3	7	$1,672.25 ($1,177.42)	$17,946.94 ($27,848.40)
			75-84	7,470	270	3.6%	3	7	$1,611.48 ($1,355.88)	$15,291.40 ($20,591.13)
			≥85	8,601	162	1.9%	3	8	$1,244.72 ($854.20)	$11,781.83 ($14,037.36)
		ODx (N=12,480)	50-64	3,102	647	20.9%	4	11	$2,251.55 ($1,586.70)	$39,543.23 ($55,400.46)
			65-74	2,807	436	15.5%	4	10	$2,005.13 ($1,975.85)	$28,615.00 ($36,559.30)
			75-84	3,251	374	11.5%	3	11	$1,907.90 ($1,330.64)	$27,883.48 ($39,282.07)
			≥85	3,320	163	4.9%	3	10	$1,583.58 ($1,039.24)	$20,740.64 ($26,802.12)
2017/2018	Present	MRDx (N=12,021)	50-64	1,812	530	29.2%	5	9	$2,479.00 ($4,447.52)	$29,231.32 ($38,635.72)
			65-74	2,385	538	22.6%	5	11	$2,216.45 ($1,482.53)	$30,678.46 ($36,923.98)
			75-84	3,397	508	15.0%	4	10	$1,921.66 ($1,312.70)	$26,089.29 ($46,716.83)
			≥85	4,427	389	8.8%	3	10	$1,603.02 ($1,087.23)	$16,740.89 ($17,077.68)
		ODx (N=46,139)	50-64	7,951	2,666	33.5%	5	11	$2,559.66 ($1,809.53)	$39,891.57 ($64,704.55)
			65-74	11,580	3,023	26.1%	5	11	$2,250.14 ($1,504.90)	$34,959.03 ($49,984.97)
			75-84	14,373	2,772	19.3%	4	11	$1,998.91 ($1,568.41)	$32,919.60 ($60,653.37)
			≥85	12,235	1,350	11.0%	4	11	$1,698.48 ($1,233.88)	$24,438.88 ($43,218.16)
	Absent	MRDx (N=29,057)	50-64	6,176	444	7.2%	3	7	$1,914.22 ($1,606.13)	$18,299.26 ($31,032.36)
			65-74	6,134	361	5.9%	3	7	$1,712.85 ($1,233.72)	$18,305.97 ($31,485.05)
			75-84	7,800	293	3.8%	3	8	$1,590.42 ($1,218.47)	$18,400.66 ($30,983.71)
			≥85	8,947	154	1.7%	2	8	$1,357.76 ($1,013.31)	$15,093.28 ($23,904.48)

		ODx (N=12,994)	50-64	3,193	664	20.8%	4	10	$2,309.61 ($1,481.58)	$36,529.85 ($56,742.15)
			65-74	3,000	436	14.5%	4	10	$2,302.18 ($2,114.59)	$35,379.00 ($50,747.41)
			75-84	3,327	316	9.5%	3	11	$1,802.58 ($1,084.04)	$26,155.76 ($30,546.26)
			≥85	3,474	161	4.6%	3	9	$1,914.79 ($1,307.81)	$22,782.09 ($29,698.66)
2018/2019	Present	MRDx (N=11,467)	50-64	1,857	529	28.5%	5	10	$2,431.22 ($1,858.09)	$34,468.89 ($42,293.78)
			65-74	2,301	517	22.5%	5	10	$2,053.14 ($1,285.05)	$29,482.20 ($39,605.62)
			75-84	3,180	514	16.2%	4	11	$1,904.32 ($1,250.60)	$28,262.62 ($40,550.58)
			≥85	4,129	350	8.5%	4	11	$1,537.86 ($1,071.96)	$25,949.64 ($70,718.41)
		ODx (N=44,608)	50-64	8,023	2,725	34.0%	5	11	$2,601.09 ($2,074.57)	$41,839.16 ($58,293.89)
			65-74	11,466	3,061	26.7%	5	11	$2,258.20 ($1,489.29)	$37,232.93 ($67,820.33)
			75-84	13,717	2,665	19.4%	4	11	$2,031.28 ($1,411.95)	$34,639.84 ($61,729.12)
			≥85	11,402	1,238	10.9%	4	11	$1,741.29 ($1,383.45)	$26,073.95 ($42,085.72)
	Absent	MRDx (N=27,292)	50-64	6,293	493	7.8%	3	7	$1,969.66 ($1,458.66)	$22,082.92 ($34,357.27)
			65-74	6,053	360	5.9%	3	8	$1,586.58 ($1,113.19)	$16,797.06 ($25,930.36)
			75-84	7,096	286	4.0%	3	8	$1,667.30 ($1,230.49)	$17,362.88 ($26,783.23)
			≥85	7,850	160	2.0%	2	8	$1,474.23 ($1,430.95)	$13,438.01 ($15,393.63)
		ODx (N=12,720)	50-64	3,182	603	19.0%	4	11	$2,454.14 ($1,667.17)	$34,902.43 ($43,119.58)
			65-74	2,935	437	14.9%	4	10	$2,220.14 ($1,675.02)	$32,856.57 ($50,708.57)
			75-84	3,273	328	10.0%	3	10	$1,975.19 ($1,290.20)	$27,692.54 ($44,568.48)
			≥85	3,330	178	5.3%	3	9	$1,856.61 ($1,745.90)	$21,073.90 ($23,532.19)

Notes: MRDx: most responsible diagnosis; ODx: other than most responsible diagnosis

Supplementary Table S6. Length of stay and total cost of hospitalization for all-cause community acquired pneumonia, by comorbidity, case coding and age group, fiscal year 2009-2018, Canada (excluding Quebec).

Fiscal Year	Comorbidity	Case coding	Age group	Number of cases	Length of stay - median (days)	Daily cost of hospital stay - average (standard deviation)	Total cost of hospital stay - average (standard deviation)
2014/2015	Present	MRDx	50-64	1,506	6	$1,721.19 ($1,304.94)	$13,724.37 ($22,310.69)
			65-74	1,920	6	$1,636.84 ($1,267.78)	$13,059.69 ($20,847.22)
			75-84	3,165	7	$1,395.90 ($1,093.81)	$11,802.54 ($21,413.25)
			≥85	4,018	8	$1,230.91 ($968.94)	$11,197.28 ($15,276.39)
		ODx	50-64	6,227	6	$1,869.81 ($1,524.03)	$17,945.43 ($46,035.23)
			65-74	9,540	7	$1,631.40 ($1,292.66)	$15,396.24 ($31,166.90)
			75-84	13,663	7	$1,445.87 ($1,189.47)	$13,840.80 ($24,765.78)
			≥85	11,207	8	$1,293.38 ($1,088.76)	$12,600.75 ($18,087.91)
	Absent	MRDx	50-64	5,494	4	$1,547.36 ($1,176.16)	$6,812.42 ($12,512.61)
			65-74	5,379	5	$1,419.70 ($1,096.77)	$6,997.78 ($12,635.92)
			75-84	7,510	5	$1,310.47 ($1,073.90)	$7,157.52 ($11,596.94)
			≥85	8,678	6	$1,184.23 ($974.45)	$8,036.77 ($13,565.37)
		ODx	50-64	2,402	7	$1,688.05 ($1,434.62)	$16,092.10 ($26,175.57)
			65-74	2,269	8	$1,482.57 ($1,226.27)	$14,961.28 ($23,798.20)
			75-84	2,676	9	$1,360.05 ($1,178.20)	$14,012.47 ($20,284.48)
			≥85	2,858	10	$1,236.48 ($1,207.05)	$14,364.40 ($20,122.74)
2015/2016	Present	MRDx	50-64	1,736	6	$1,839.49 ($1,462.37)	$14,985.50 ($23,428.30)
			65-74	2,013	6	$1,667.52 ($1,394.71)	$13,283.80 ($23,372.40)
			75-84	3,120	7	$1,452.25 ($1,116.17)	$12,069.98 ($16,913.53)
			≥85	3,684	8	$1,248.72 ($960.83)	$11,671.53 ($17,301.86)
		ODx	50-64	7,615	6	$1,984.93 ($1,646.74)	$20,137.81 ($39,747.92)
			65-74	10,608	7	$1,718.20 ($1,435.94)	$17,742.95 ($39,013.86)
			75-84	13,601	7	$1,509.78 ($1,187.95)	$15,042.86 ($26,645.01)
			≥85	11,251	8	$1,336.47 ($1,069.15)	$13,265.27 ($24,415.88)
	Absent	MRDx	50-64	6,158	4	$1,577.68 ($1,201.85)	$6,999.10 ($10,841.42)
			65-74	5,488	4	$1,485.31 ($1,165.92)	$6,940.17 ($11,283.78)
			75-84	7,063	5	$1,337.17 ($1,059.32)	$7,333.89 ($11,304.04)
			≥85	7,696	6	$1,214.56 ($998.06)	$8,153.02 ($13,566.27)
		ODx	50-64	3,132	7	$1,803.35 ($1,485.68)	$18,045.74 ($32,125.86)
			65-74	2,663	8	$1,598.84 ($1,393.40)	$15,684.44 ($25,217.61)
			75-84	3,068	8	$1,422.34 ($1,270.44)	$15,047.49 ($27,451.48)
			≥85	3,043	9	$1,319.81 ($1,264.98)	$14,067.69 ($18,352.67)
2016/2017	Present	MRDx	50-64	1,688	6	$1,885.11 ($1,578.35)	$15,733.69 ($26,983.55)
			65-74	2,153	6	$1,717.99 ($1,532.66)	$13,638.17 ($21,349.16)
			75-84	3,261	7	$1,481.68 ($1,138.03)	$12,359.67 ($19,842.93)
			≥85	4,226	8	$1,305.63 ($1,056.29)	$10,727.28 ($12,875.89)

		ODx	50-64	7,604	7	$1,944.56 ($1,539.65)	$20,052.05 ($39,786.23)
			65-74	10,959	7	$1,748.28 ($1,359.11)	$17,555.17 ($35,917.82)
			75-84	14,189	7	$1,540.77 ($1,246.51)	$14,895.88 ($30,088.98)
			≥85	11,890	8	$1,347.49 ($1,122.90)	$12,914.46 ($20,513.49)
	Absent	MRDx	50-64	5,738	4	$1,587.35 ($1,246.28)	$7,042.45 ($16,824.30)
			65-74	5,906	4	$1,491.85 ($1,166.80)	$6,963.37 ($11,622.93)
			75-84	7,470	5	$1,364.97 ($1,087.17)	$7,378.42 ($13,537.54)
			≥85	8,601	6	$1,236.07 ($985.05)	$7,694.43 ($11,162.14)
		ODx	50-64	3,102	7	$1,808.84 ($1,584.05)	$18,825.08 ($34,316.90)
			65-74	2,807	7	$1,636.07 ($1,533.71)	$15,866.19 ($22,093.74)
			75-84	3,251	8	$1,444.64 ($1,247.76)	$14,757.08 ($22,597.76)
			≥85	3,320	9	$1,293.79 ($1,176.67)	$14,403.79 ($19,025.05)
2017/2018	Present	MRDx	50-64	1,812	6	$1,960.19 ($2,733.37)	$14,501.72 ($24,164.07)
			65-74	2,385	6	$1,762.88 ($1,321.99)	$14,033.85 ($21,677.27)
			75-84	3,397	7	$1,523.33 ($1,233.15)	$12,082.01 ($21,986.01)
			≥85	4,427	8	$1,340.70 ($1,140.07)	$11,108.37 ($13,540.38)
		ODx	50-64	7,951	6	$2,060.34 ($1,654.39)	$20,246.61 ($41,666.43)
			65-74	11,580	7	$1,790.77 ($1,454.56)	$16,976.21 ($30,572.21)
			75-84	14,373	7	$1,571.55 ($1,325.33)	$15,496.81 ($32,157.12)
			≥85	12,235	8	$1,396.88 ($1,157.50)	$13,429.76 ($21,161.76)
	Absent	MRDx	50-64	6,176	4	$1,646.91 ($1,295.71)	$6,832.04 ($10,496.26)
			65-74	6,134	4	$1,513.67 ($1,185.10)	$6,973.98 ($10,495.47)
			75-84	7,800	5	$1,391.90 ($1,124.61)	$7,431.26 ($11,606.47)
			≥85	8,947	6	$1,274.39 ($1,062.38)	$7,832.42 ($11,368.13)
		ODx	50-64	3,193	7	$1,888.01 ($1,563.22)	$17,916.89 ($31,903.04)
			65-74	3,000	7	$1,646.25 ($1,473.52)	$16,194.82 ($26,001.68)
			75-84	3,327	8	$1,471.78 ($1,243.44)	$15,131.32 ($26,076.49)
			≥85	3,474	9	$1,342.64 ($1,246.23)	$13,925.39 ($19,043.59)
2018/2019	Present	MRDx	50-64	1,857	6	$1,912.71 ($1,571.20)	$15,821.86 ($26,186.46)
			65-74	2,301	6	$1,714.87 ($1,264.60)	$14,450.42 ($26,142.72)
			75-84	3,180	7	$1,544.89 ($1,242.08)	$13,118.43 ($20,599.69)
			≥85	4,129	8	$1,321.53 ($1,068.66)	$12,040.72 ($24,335.70)
		ODx	50-64	8,023	7	$2,066.88 ($1,744.06)	$20,876.27 ($39,159.70)
			65-74	11,466	7	$1,813.77 ($1,455.29)	$17,919.41 ($39,864.92)
			75-84	13,717	7	$1,617.39 ($1,279.52)	$16,078.68 ($32,614.42)
			≥85	11,402	8	$1,407.16 ($1,213.96)	$13,964.14 ($21,616.16)
	Absent	MRDx	50-64	6,293	4	$1,657.31 ($1,298.13)	$7,372.90 ($12,705.08)
			65-74	6,053	4	$1,517.31 ($1,184.41)	$6,940.08 ($10,379.49)
			75-84	7,096	5	$1,441.90 ($1,185.71)	$7,374.10 ($11,792.05)
			≥85	7,850	6	$1,306.37 ($1,099.42)	$7,894.14 ($10,717.33)

		ODx	50-64	3,182	7	$1,898.97 ($1,550.50)	$18,028.12 ($28,990.44)
			65-74	2,935	8	$1,699.59 ($1,568.40)	$16,307.77 ($26,396.27)
			75-84	3,273	8	$1,489.30 ($1,308.78)	$16,088.98 ($25,686.36)
			≥85	3,330	8	$1,393.78 ($1,368.24)	$13,608.58 ($16,804.42)

Notes: MRDx: most responsible diagnosis; ODx: other than most responsible diagnosis
